# Epitope-diverse autoantibodies induce neutrophil–epithelial niches driving kidney injury in ANCA-associated glomerulonephritis

**DOI:** 10.1084/jem.20260506

**Published:** 2026-10-19

**Authors:** Masayuki Nishide, Hiroaki Matsushita, Yumiko Mizuno, Kei Nishimura, Patrick M. Lelliott, Shoji Kawada, Takashi Fujii, Shogo Kamikawaji, Tatsuya Nonaka, Daisuke Motooka, Shoichi Metsugi, Hiroshi Shimagami, Atsuko Tsujii Miyamoto, Yasuhiro Kato, Kohei Tsujimoto, Ryuya Edahiro, Yuya Shirai, Yuji Yamamoto, Yujiro Naito, Hachiro Konaka, Mayuko Izumi, Maiko Naito, Yoshimitsu Nakanishi, Tomoko Namba-Hamano, Kazunori Inoue, Atsushi Takahashi, Masayuki Mizui, Shigeaki Nakazawa, Yoichi Kakuta, Kenji Funakoshi, Makoto Terada, Machiko Irie, Takuya Torizawa, Ryusuke Omiya, Nicolas I. Smith, Yoshitaka Isaka, Norio Nonomura, Yukinori Okada, Kunihiro Hattori, Masashi Narazaki, Atsushi Kumanogoh

**Affiliations:** 1 Department of Respiratory Medicine and Clinical Immunology, Graduate School of Medicine, The University of Osaka, Suita, Osaka, Japan; 2 Department of Immunopathology, World Premier International Research Center Initiative (WPI), Immunology Frontier Research Center (IFReC), The University of Osaka, Suita, Osaka, Japan; 3 Department of Advanced Clinical and Translational Immunology, Graduate School of Medicine, The University of Osaka, Suita, Osaka, Japan; 4 Research Division, Chugai Pharmaceutical Co., Ltd., Gotemba, Shizuoka, Japan; 5 Joint Research Chair of Innovative Drug Discovery in Immunology, World Premier International Research Center Initiative (WPI), Immunology Frontier Research Center (IFReC), The University of Osaka, Suita, Osaka, Japan; 6 Biophotonics Laboratory, World Premier International Research Center Initiative (WPI), Immunology Frontier Research Center (IFReC), The University of Osaka, Suita, Osaka, Japan; 7 NGS Core Facility, Bioinformatics Center, Research Institute for Microbial Diseases, The University of Osaka, Suita, Osaka, Japan; 8 Department of Statistical Genetics, Graduate School of Medicine, The University of Osaka, Suita, Osaka, Japan; 9 Laboratory for Systems Genetics, RIKEN Center for Integrative Medical Sciences, Yokohama, Japan; 10 Integrated Frontier Research for Medical Science Division, Institute for Open and Transdisciplinary Research Initiatives (OTRI), The University of Osaka, Suita, Osaka, Japan; 11 Department of Nephrology, Graduate School of Medicine, The University of Osaka, Suita, Osaka, Japan; 12 Department of Urology, Graduate School of Medicine, The University of Osaka, Suita, Osaka, Japan; 13 Itami City Hospital, Itami, Japan; 14 Department of Statistical Immunology, World Premier International Research Center Initiative (WPI), Immunology Frontier Research Center (IFReC), The University of Osaka, Suita, Osaka, Japan; 15 Department of Genome Informatics, Graduate School of Medicine, The University of Tokyo, Tokyo, Japan; 16 Japan Agency for Medical Research and Development – Core Research for Evolutional Science and Technology (AMED–CREST), The University of Osaka, Suita, Osaka, Japan; 17 Center for Infectious Diseases for Education and Research (CiDER), The University of Osaka, Suita, Osaka, Japan; 18 Center for Advanced Modalities and DDS (CAMaD), The University of Osaka, Suita, Osaka, Japan

## Abstract

Autoantibody-mediated inflammation drives tissue injury in autoimmune diseases, yet how pathogenic antibodies evoke local immune activation remains poorly understood. Here, we show that spatially organized neutrophil–epithelial niches contribute to kidney injury in myeloperoxidase (MPO)-antineutrophil cytoplasmic antibody–associated glomerulonephritis. Single-cell–resolved spatial transcriptomics of treatment-naïve patient kidney biopsies identified early-phase inflammatory niches enriched with HAVCR1^+^ injured proximal tubular epithelial cells, neutrophils, lymphocytes, and TREM2^+^ macrophages. Injured epithelial cells expressed chemokines including CXCL6 and CXCL16, defining a microenvironment associated with local immune cell accumulation. Neutrophils within these niches were characterized by transcriptional programs enriched for type II interferon and Fcγ receptor (FcγR)-related signaling. Mechanistically, combinations of monoclonal anti-MPO antibodies recognizing distinct epitopes enhanced neutrophil activation and induced pauci-immune crescentic glomerulonephritis in mice, recapitulating key aspects of epithelial injury observed in human kidneys. One-armed anti-MPO antibodies engineered to minimize FcγR engagement ameliorated kidney injury in mice. Together, these findings identify pathogenic neutrophil–epithelial niche formation as a driver of autoimmune kidney injury.

## Teaser

Nishide et al. identify neutrophil–epithelial niches associated with local kidney injury in ANCA-associated glomerulonephritis using single-cell–resolved spatial transcriptomics. Neutrophils in these niches show Fcγ receptor-related programs linked to epitope-diverse autoantibodies, while anti-MPO antibody combinations reproduce key pathological features in mice and engineered one-armed antibodies attenuate kidney injury in vivo.

## Graphical Abstract

**Figure:**
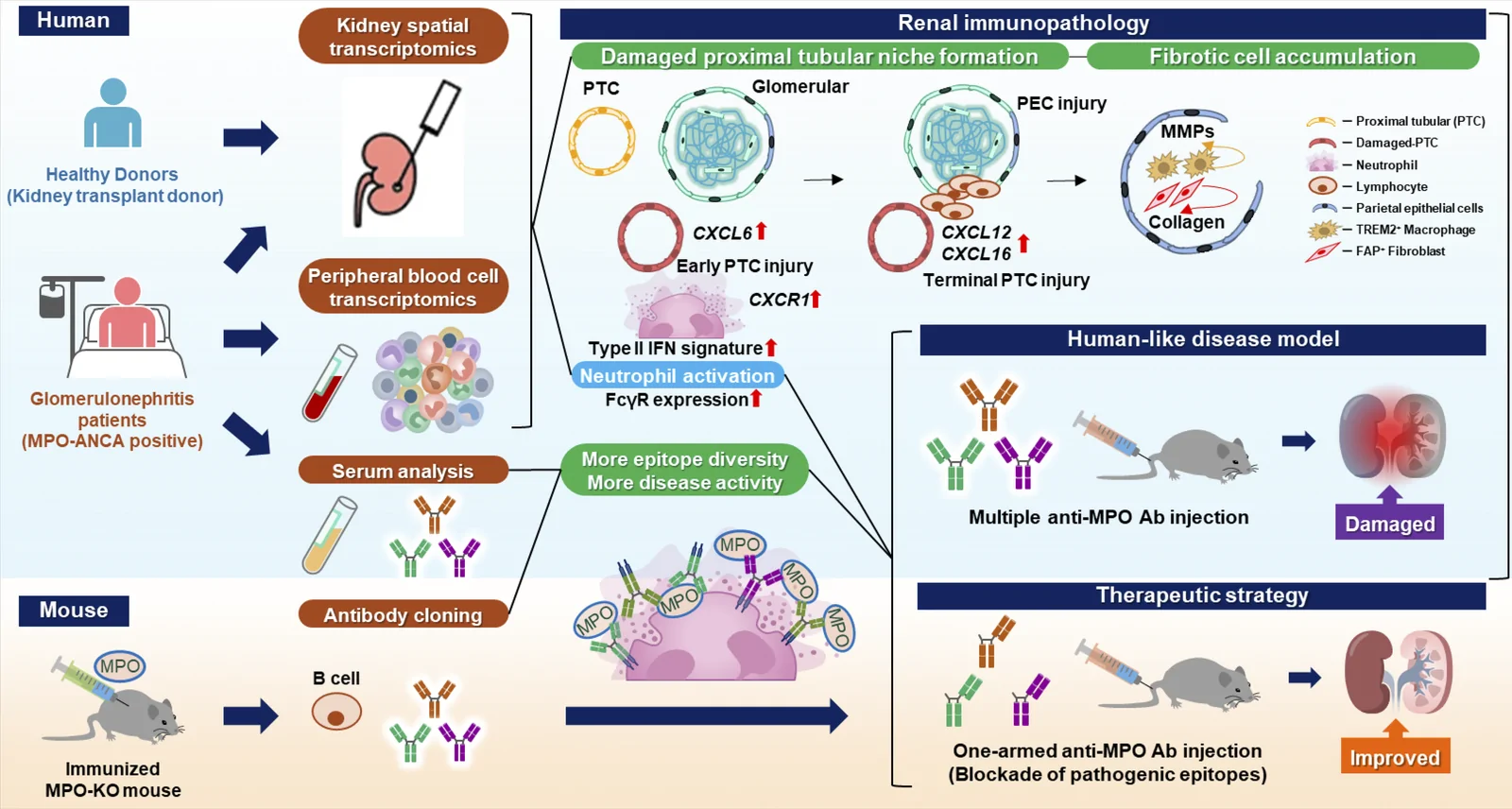

## Introduction

Autoantibody-driven inflammation is a fundamental mechanism underlying organ-specific autoimmune diseases. Pathogenic antibodies trigger immune activation and tissue injury across multiple organs (Anders et al., 2023; Huang et al., 2023b; Lee and Kalluri, 2010). Despite advances in defining systemic immune dysregulation, how pathogenic autoantibodies initiate local tissue injury within affected organs remains incompletely understood.

Recent spatial profiling studies of human tissues have revealed complex interactions between immune cells and parenchymal cells that accompany tissue remodeling and fibrosis (Krebs et al., 2021; Ma et al., 2025; Melo Ferreira et al., 2021). However, these studies typically capture established inflammatory lesions rather than the onset of disease, leaving the early events that initiate tissue injury largely unresolved (Daehn and Duffield, 2021). Addressing this challenge will require studying early-stage disease with mechanistic investigation to define how antibody-driven immune activation organizes into pathogenic cellular niches within tissues (Nishide et al., 2024).

Autoimmune nephritides provide a clinically relevant system to investigate antibody-mediated tissue injury. Among these diseases, antineutrophil cytoplasmic antibody (ANCA)–associated glomerulonephritis arises as a primary renal manifestation rather than a secondary consequence of systemic inflammation, providing a well-defined context to dissect immune–tissue interactions (Foresto-Neto et al., 2024; Herrington et al., 2026; Kant et al., 2022; Sultana et al., 2025). Pathogenic autoantibodies against myeloperoxidase (MPO) or proteinase 3 activate neutrophils and induce effector responses including ROS production and neutrophil extracellular trap (NET) formation (Kitching et al., 2020). Anti-MPO antibodies recognize multiple epitopes on the MPO molecule, raising the possibility that epitope diversity may shape neutrophil activation during disease (Gou et al., 2013; Roth et al., 2013; Suzuki et al., 2007). Understanding how pathogenic immune responses are organized within tissues is essential not only for defining the mechanisms that initiate organ damage but also for enabling mechanism-based therapeutic interventions.

In this study, we combine single-cell–resolved spatial transcriptomics with mechanistic modeling to investigate how pathogenic autoantibodies initiate kidney injury in autoimmune glomerulonephritis. Spatial profiling of kidney biopsies from new-onset, treatment-naïve patients with MPO-ANCA–associated glomerulonephritis identifies peri-glomerular inflammatory niches composed of injured proximal tubular epithelial cells and infiltrating immune populations. Mechanistic experiments demonstrate that combinations of anti-MPO antibodies recognizing distinct epitopes enhance neutrophil activation through Fcγ receptor (FcγR) signaling and induce crescentic glomerulonephritis *in vivo*. Together, these findings reveal a spatially organized neutrophil–epithelial circuit linking epitope-diverse autoantibody recognition to the initiation of kidney injury.

## Results

### Single-cell–resolution spatial transcriptomics of kidney biopsies from treatment-naïve patients with ANCA-associated glomerulonephritis

**Figure 1.**
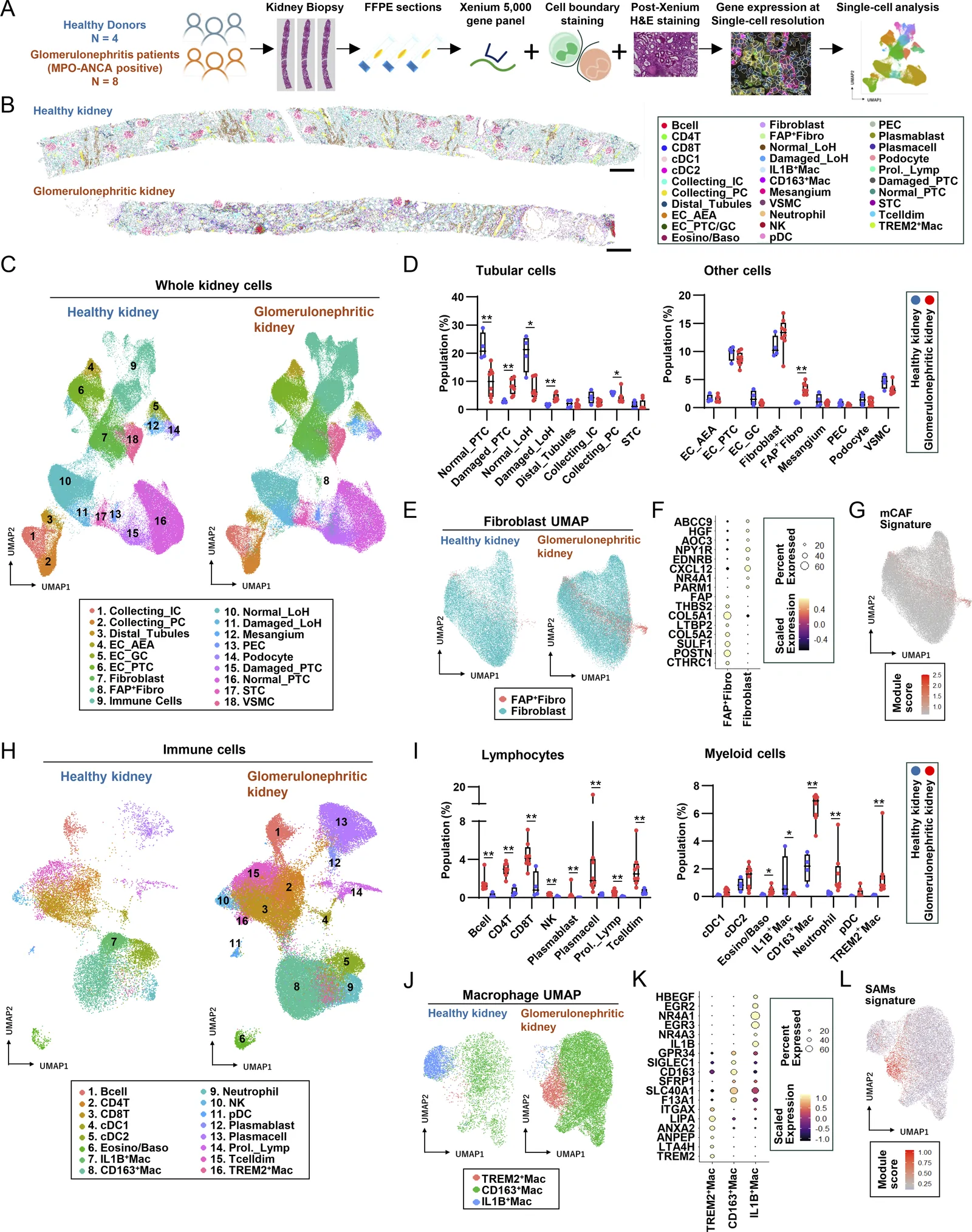
**Single-cell–resolution spatial omics profiling of cellular reorganization in treatment-naïve ANCA-associated glomerulonephritis. (A)** Experimental workflow. Renal needle biopsy specimens were obtained from healthy donors (*n* = 4) and newly diagnosed, treatment-naïve patients with MPO-ANCA–positive glomerulonephritis (*n* = 8). FFPE kidney sections were analyzed using the 10x Genomics Xenium platform with a targeted 5,000-gene panel. **(B)** Representative spatial cell type maps of kidney sections obtained from a healthy donor and a patient with glomerulonephritis. Cell boundaries were delineated based on nuclear and cytoplasmic/membrane staining. Cells are colored by annotated cell type (abbreviations shown; see Fig. S1 A for full definitions). Scale bar, 200 μm. **(C)** UMAPs of all kidney tissue cells from healthy donors (left) and patients with glomerulonephritis (right), colored based on cell type annotation. **(D)** Relative proportions of each kidney tissue cell population in healthy donors (blue) and patients with glomerulonephritis (red). Boxes indicate the interquartile range (Q1–Q3), the line marks the median; whiskers span the minimum to maximum. *P < 0.05; **P < 0.01; nominal P values were calculated using a two-sided Mann–Whitney U test. **(E)** UMAPs of kidney fibroblasts from healthy donors (left) and patients with glomerulonephritis (right). **(F)** Balloon plots showing representative marker gene expression in each fibroblast population. Balloon color indicates the average of the scaled expression of each gene, and balloon size indicates the proportion of cells expressing the indicated genes. **(G)** Module scoring of fibroblast subsets using a mCAF gene signature. Cells are colored by scaled module score. **(H)** UMAPs of immune cells from healthy donors (left) and patients (right), colored based on cell type annotation. **(I)** Relative proportions of immune cell populations in healthy donors (blue) and patients with glomerulonephritis (red). Boxplots and P values are shown as in D. **(J)** UMAPs of kidney macrophages from healthy donors (left) and patients (right). **(K)** Balloon plots showing representative marker gene expression in each macrophage population. Balloon color and size are defined as in F. **(L)** Module scoring of macrophage subsets using a SAM gene signature. Cells are colored by scaled module score.

**Figure S1.**
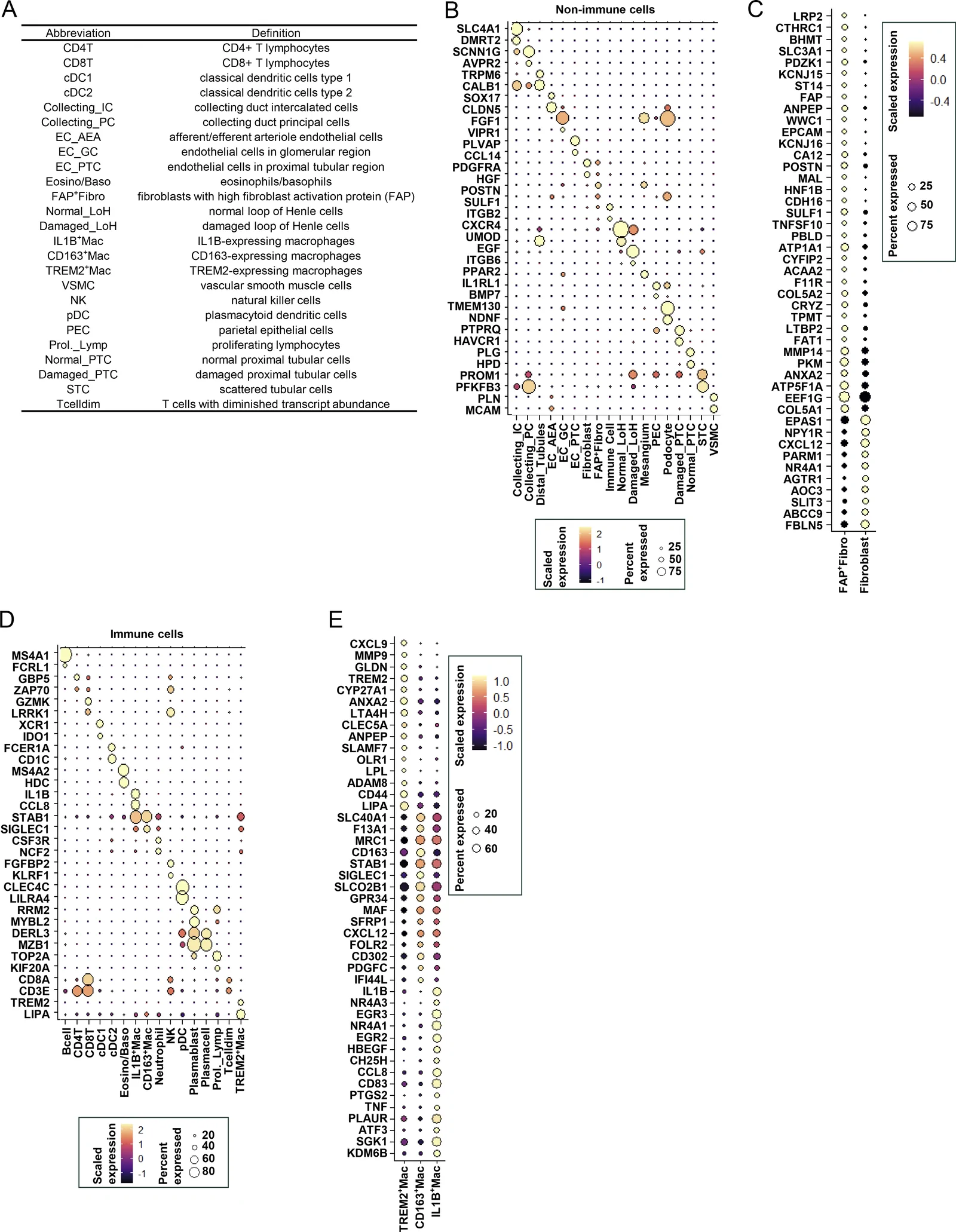
**Gene expression profiles of human kidney tissues, related to Fig. 1. (A)** Abbreviations and definitions of human renal cell clusters. **(B)** Balloon plot showing highly expressed genes in nonimmune cell populations within kidney tissues. Balloon color indicates the average of the scaled expression of the indicated genes. Balloon size indicates the percentage of cells expressing the indicated genes. **(C)** Balloon plot showing highly expressed genes in fibroblast cell populations. **(D)** Balloon plot showing highly expressed genes in immune cell populations. **(E)** Balloon plot showing highly expressed genes in macrophage cell populations.

Kidney needle biopsy specimens were obtained from four healthy kidney donors and eight newly diagnosed, treatment-naïve patients with MPO-ANCA–positive microscopic polyangiitis (MPA). Healthy donor samples were collected during donor nephrectomy for renal transplantation. Detailed clinical information is provided in Table S1. Formalin-fixed, paraffin-embedded (FFPE) samples were subjected to spatial transcriptomic profiling using the 10x Genomics Xenium platform (Fig. 1 A). Cell boundaries were determined using nuclear staining and cytoplasmic/membrane protein immunostaining. Using a targeted 5,000-gene panel, individual cells were annotated, and a comprehensive cell type map of the kidney tissue was constructed. Representative cell type maps from a healthy donor and a patient are shown in Fig. 1 B, and donor-specific maps are provided in the deposited Figshare dataset described in the Data availability section. The cell type abbreviations used for annotation are listed in Fig. S1 A. We identified 32 distinct cell populations, including tubular epithelial cells, glomerular structural cells, interstitial fibroblasts, and hematopoietic immune cells.

We first examined the cellular composition of kidney tissue, focusing on non-hematopoietic cell populations. Integrated uniform manifold approximation and projection (UMAPs) of kidney cells from healthy donors and patients are shown in Fig. 1 C, and representative marker gene expression patterns for each cluster are shown in Fig. S1 B. The UMAPs for individual participants are shown in the deposited Figshare dataset described in the Data availability section. Compared with healthy donor kidneys, patient kidneys showed a significant increase in damaged proximal tubular cells (Damaged_PTCs), characterized by high HAVCR1 expression (Lake et al., 2023), and damaged loop of Henle cells (Damaged_LoH), characterized by high ITGB6 expression (Lake et al., 2023) (Fig. 1 D). In contrast, normal PTCs (Normal_PTCs), normal loop of Henle cells, and collecting duct principal cells were significantly reduced in the kidneys of the patients. In addition, fibroblasts expressing fibroblast activation protein (FAP^+^ fibroblasts) (You et al., 2023) were significantly increased in patient kidneys (Fig. 1 D). Fibroblast-focused analysis identified FAP^+^ fibroblasts as a distinct population in patient kidneys (Fig. 1 E), showing elevated expression of extracellular matrix–associated genes, such as *COL5A1/2* and *POSTN* (Fig. 1 F). An expanded panel of representative marker gene expression patterns across fibroblast clusters is shown in Fig. S1 C. Highly expressed genes identified by differentially expressed gene (DEG) analysis of FAP^+^ fibroblasts are listed in Table S2. Module scoring analysis revealed similarity between the FAP^+^ fibroblast subset and matrix cancer–associated fibroblasts (mCAFs) (Fig. 1 G), a pro-tumor fibroblast phenotype characterized by enhanced extracellular matrix production and tissue remodeling (Cords et al., 2023; Yoon et al., 2021).

Next, we focused on the immune cells and performed further dimensionality reduction to refine their annotations. The UMAPs of immune cells from healthy donors and patients are shown in Fig. 1 H, and representative marker gene expression patterns for each cluster are shown in Fig. S1 D. The UMAPs for individual participants are shown in the deposited Figshare dataset described in the Data availability section. Compared with healthy donors, patient kidneys showed a broad expansion of immune cell populations, including B cells, CD4^+^ and CD8^+^ T cells, natural killer cells, plasmablasts, plasma cells, and granulocytes (Fig. 1 I). Among the macrophage subsets, *IL1B*^+^ macrophages were reduced, whereas *CD163*^+^ macrophages and macrophages expressing triggering receptor expressed on myeloid cells 2 (TREM2^+^ macrophages) were significantly increased in patient kidneys (Fig. 1 I). Macrophage-focused analysis identified TREM2^+^ macrophages as a distinct population enriched in patient kidneys (Fig. 1 J). TREM2^+^ macrophages showed elevated expression of *LIPA* and *ANXA2* (Fig. 1 K), consistent with macrophage programs related to lipid handling and tissue remodeling (Jaitin et al., 2019). An expanded panel of representative marker gene expression patterns across macrophage clusters is shown in Fig. S1 E. Highly expressed genes identified by DEG analysis of each macrophage subset are listed in Table S2. Module scoring analysis revealed similarities between TREM2^+^ macrophages and scar-associated macrophages (SAMs) (Fig. 1 L), a profibrotic macrophage population associated with tissue remodeling and fibrosis in chronically injured organs (Fabre et al., 2023; Ramachandran et al., 2019). Together, these data demonstrate the extensive reorganization of renal cell composition in MPO-ANCA–associated glomerulonephritis. Patient kidneys are characterized by the expansion of damaged epithelial populations and fibrogenic subsets, including FAP^+^ fibroblasts and TREM2^+^ macrophages, which exhibit transcriptional programs shared with other fibrotic disease contexts.

### Spatial niche analysis identifies expanded injury- and immune-associated microenvironments in ANCA-associated glomerulonephritis

**Figure 2.**
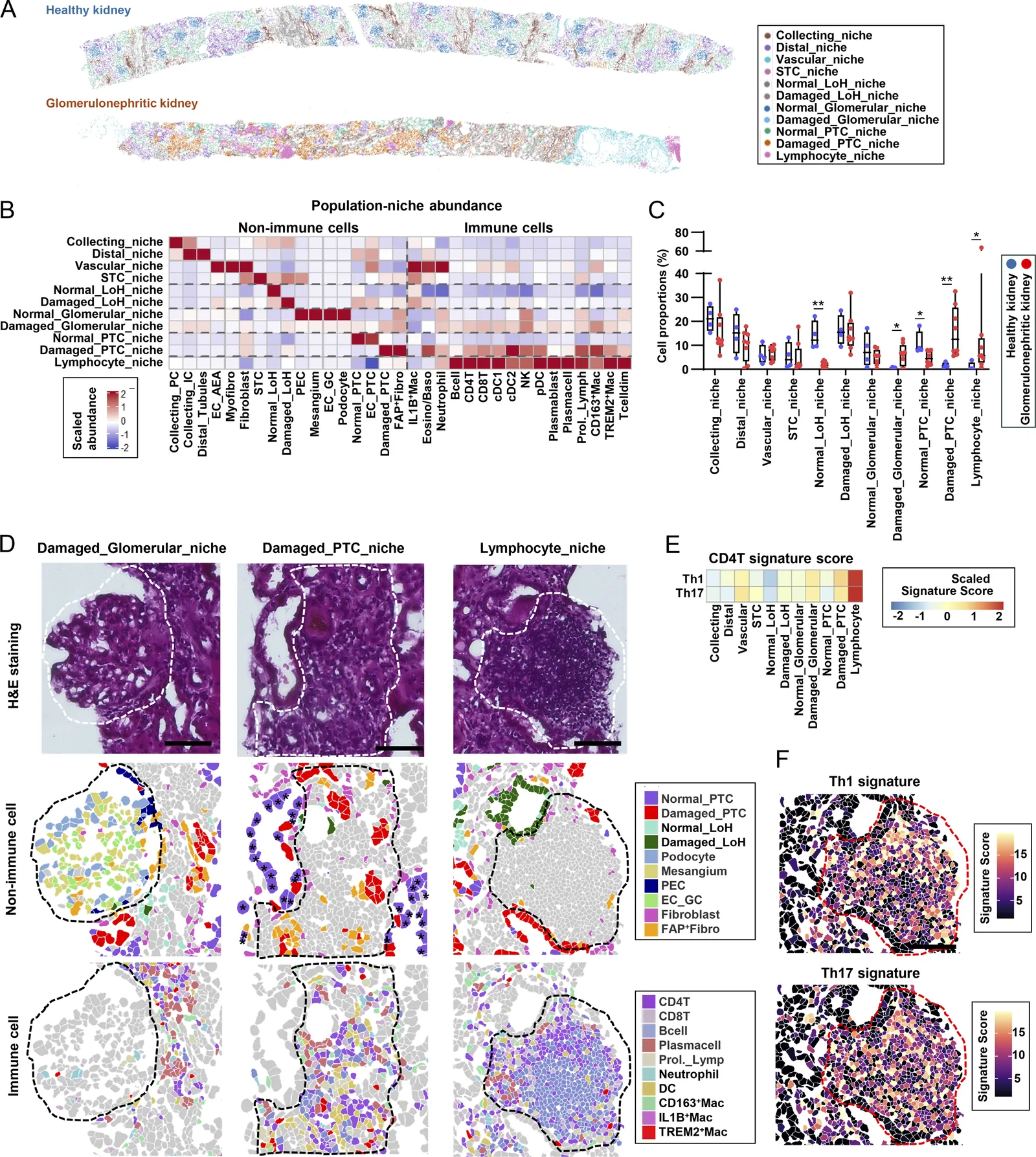
**Spatial niche analysis identifies disease-associated niches in ANCA-associated glomerulonephritis. (A)** Representative spatial transcriptomic–based niche maps from a healthy donor and a patient with glomerulonephritis. 11 cellular niches were identified based on spatial proximity analysis of transcriptomic data. Scale bar, 200 μm. **(B)** Heatmap showing the enrichment scores for each cell subpopulation across eleven proximity-informed niches. Niches were annotated based on the composition of neighboring cells, and the color scale shows the degree of enrichment in each niche. **(C)** Relative proportions of cells assigned to each niche within kidney tissue from healthy donors (blue) and patients with ANCA-associated glomerulonephritis (red). *P < 0.05; **P < 0.01; nominal P values were calculated using a two-sided Mann–Whitney U test. **(D)** Representative regions of disease-associated niches shown by H&E staining and spatial cell type annotation maps of nonimmune and immune cell populations. Each niche region is indicated by a dashed line. In the Damaged_PTC niche panel, a Normal_PTC region outside the niche is indicated by a star. Scale bar, 50 μm. **(E)** Enrichment scores of Th1- and Th17-related gene signatures across niches. **(F)** Spatial feature maps showing Th1- and Th17-related gene signatures in a representative Lymphocyte_niche. The niche region is indicated by a dashed line. Scale bar, 50 μm.

To characterize the spatial organization of renal lesions, we performed cell–cell proximity analysis using spatial transcriptomic data and identified 11 distinct cellular niches: Collecting duct niche, distal tubular cell niche, vascular niche, scattered tubular cell niche, LoH (normal/damaged) niches, glomerular cell (normal/damaged) niches, PTC (normal/damaged) niches, and lymphocyte-enriched niches. Representative niche maps from a healthy donor and a patient are shown in Fig. 2 A, and donor-specific niche maps are available in the deposited Figshare dataset described in the Data availability section. The cellular composition differed across niches. Immune cell populations were preferentially represented in the Damaged_PTC and lymphocyte-enriched niches, whereas nonimmune epithelial and stromal populations defined the corresponding tubular, glomerular, and vascular niches (Fig. 2 B). We next quantified the proportion of cells assigned to each niche within the kidney tissue. The damaged glomerular, damaged PTC, and lymphocyte-enriched niches were significantly more highly represented in patient kidneys than in healthy kidneys (Fig. 2 C). Representative regions corresponding to these niches are shown with the matched H&E-stained images and spatial maps of nonimmune and immune cell populations (Fig. 2 D). In the damaged glomerular niche, glomerular injury was accompanied by mesangial proliferation and FAP^+^ fibroblast populations. In the damaged PTC niche, injured PTCs were spatially associated with infiltrating immune cells. In the lymphocyte-enriched niche, dense lymphocytic infiltration was observed adjacent to stromal regions (Fig. 2 D). Consistent with a previous Visium-based study of ANCA-associated glomerulonephritis, this lymphocyte-enriched niche showed enrichment of Th1- and Th17-related gene signatures (Fig. 2, E and F), supporting the presence of inflammatory CD4^+^ T cell programs in renal lesions (Engesser et al., 2024).

### Glomerular niche analysis reveals a stepwise spatiotemporal program of glomerulonephritis progression

**Figure 3.**
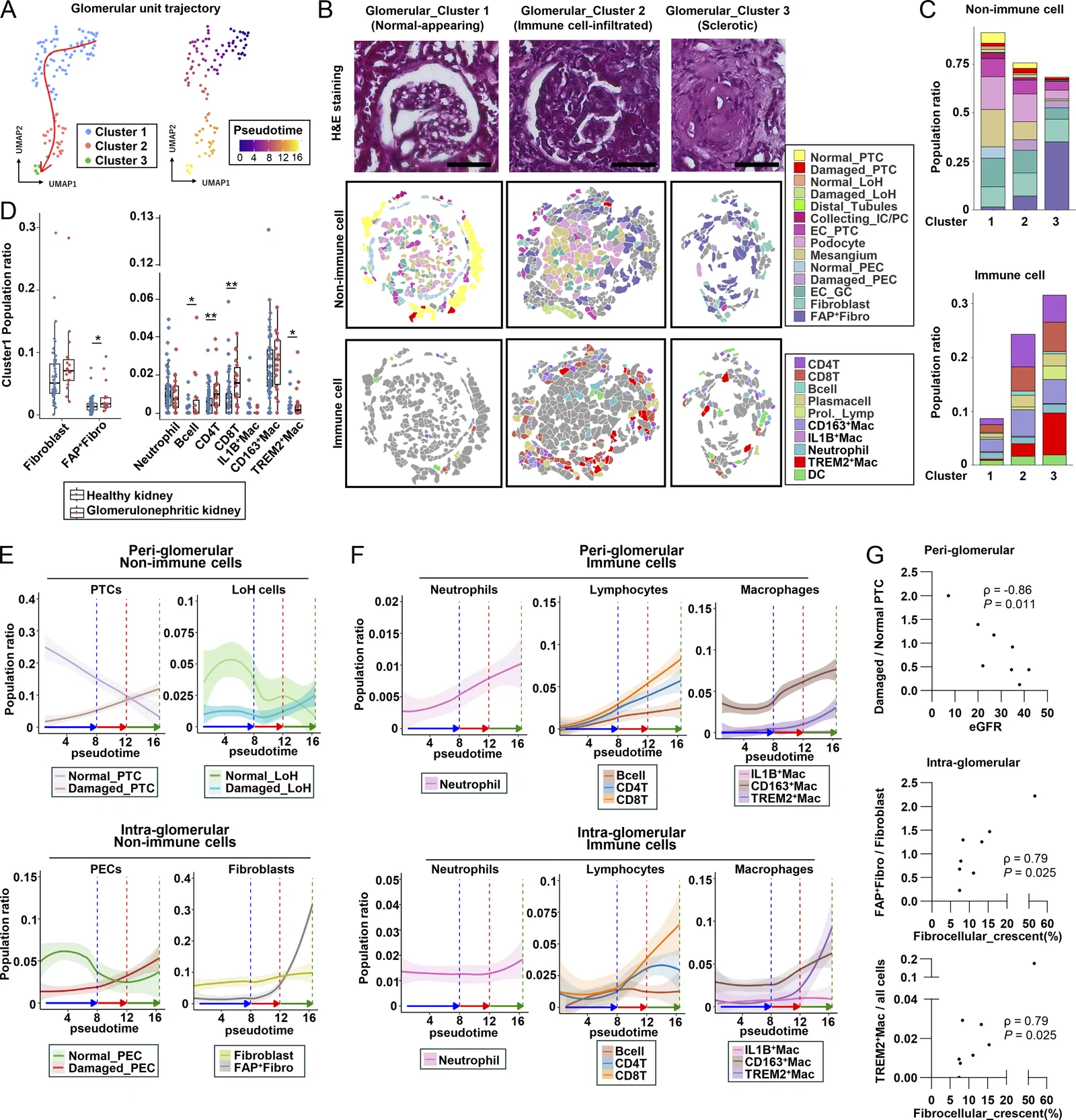
**Glomerular-level spatial trajectory analysis reveals early peri-glomerular injury and progressive glomerular remodeling. (A)** UMAP-based clustering (left) and pseudotime trajectory analysis (right) of 103 glomerular niches extracted from 12 kidney biopsy samples. Glomeruli were classified into three clusters based on pseudobulk gene expression profiles. The red arrow indicates the estimated trajectory of disease progression. **(B)** Representative histopathological features and corresponding spatial transcriptomic cell type maps for each glomerular cluster. Cluster 1, normal-appearing glomeruli; cluster 2, immune cell–infiltrated glomeruli with mesangial proliferation and minimal fibrosis; cluster 3, sclerotic glomeruli. Cells are colored by cell type annotation. Scale bar, 50 μm. **(C)** Stacked bar plots of the relative cell type composition of glomerular clusters 1–3. The bar colors correspond to the cell type annotations in B. **(D)** Relative proportions of cells assigned to cluster 1 niche from healthy donors (blue) and patients with ANCA-associated glomerulonephritis (red). *P < 0.05; **P < 0.01; nominal P values were calculated using a two-sided Mann–Whitney U test. **(E)** Sequential changes in nonimmune cell populations across pseudotime in regions surrounding glomerular niches (peri-glomerular, upper panels) and within glomerular niches (intra-glomerular, lower panels). The x-axis represents pseudotime reflecting disease progression, and the y-axis indicates the relative abundance of each cell subset. Lines show the smoothed mean with 95% confidence intervals. **(F)** Sequential changes in immune cell populations across pseudotime. **(G)** Correlation between spatially defined proportions of selected cell subsets and representative clinical parameters. Correlation coefficients (*ρ*) and P values were calculated using Spearman’s rank correlation.

To determine how coordinated changes in renal cellular composition are related to disease progression, we next focused on glomerular microenvironments. In total, 103 glomerular niches were identified and clustered based on the pseudobulk gene expression profiles of the constituent cells (Fig. 3 A). The glomerular niches were classified into three distinct clusters, and pseudotime trajectory analysis revealed a continuous shift in gene expression along the temporal axis (Fig. 3 A). The corresponding glomerular lesions were evaluated independently by renal pathologists and rheumatologists. Cluster 1 corresponded to normal-appearing glomeruli (*N* = 74), cluster 2 to immune cell–infiltrated glomeruli characterized by mesangial proliferation (*N* = 23), and cluster 3 to sclerotic glomeruli (*N* = 6). Representative glomerular regions from each cluster are shown in Fig. 3 B, with the corresponding H&E-stained images and separate spatial maps of nonimmune and immune cell populations. Analysis of the relative cell type composition of clusters 1–3 demonstrated distinct cellular profiles across the three clusters. FAP^+^ fibroblasts were more highly represented in clusters 2 and 3 than in cluster 1, and TREM2^+^ macrophages showed a similar pattern (Fig. 3 C). Of note, even within cluster 1, which comprised histologically normal-appearing glomeruli, glomerular niches from patients with ANCA-associated glomerulonephritis contained significantly higher proportions of FAP^+^ fibroblasts, B cells, T cells, and TREM2^+^ macrophages than those from healthy controls (Fig. 3 D). These findings suggest that disease-associated cellular alterations may already be present in glomeruli that remain histologically normal.

**Figure S2.**
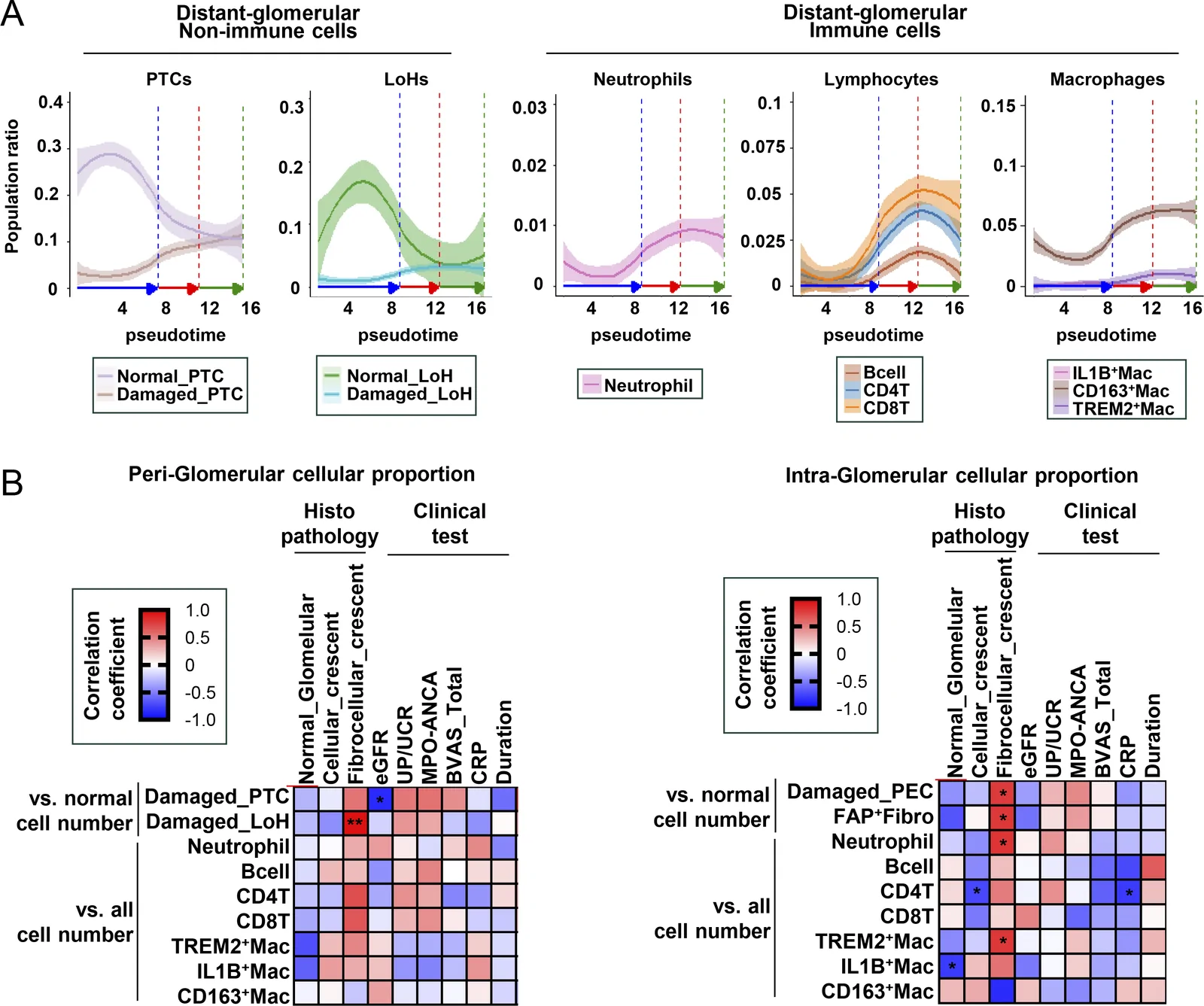
**Pseudotime trajectories in distant peri-glomerular regions and clinical correlations of glomerular-region cell populations, related to Fig. 3. (A)** Sequential changes in the indicated cell populations across pseudotime in distant peri-glomerular regions, defined as areas located two to five glomerular radii from each glomerulus. The x-axis represents pseudotime reflecting disease progression, and the y-axis indicates the relative abundance of each cell subset. Lines show the smoothed mean with 95% confidence intervals. **(B)** Heatmap showing Spearman’s correlation between clinical parameters and cell type abundances in glomerular niche–surrounding regions (peri-glomerular) and within glomerular niches (intra-glomerular). The color scale indicates correlation coefficients (ρ), and statistically significant correlations (**P < 0.05, *P < 0.1) are marked.

Analysis of nonimmune cell composition during pseudotime disease progression revealed that Damaged_PTCs were increased in the peri-glomerular regions even in the normal-appearing glomerular stage, whereas Normal_PTCs were reduced (Fig. 3 E). As the disease progressed to the immune cell–infiltrated stage, the injury extended to additional epithelial compartments, including LoH and parietal epithelial cell (PEC) populations. At the sclerotic stage, FAP^+^ fibroblasts markedly expanded within glomerular niches (Fig. 3 E). Focusing on immune cell populations, neutrophil and lymphocyte accumulation in peri-glomerular regions began at the earliest stage (Fig. 3 F). With the progression to the immune cell–infiltrated stage, CD4^+^ T cells, CD8^+^ T cells, and B cells further accumulated both within and around the glomeruli. At the sclerotic stage, TREM2^+^ macrophages markedly expanded within glomerular niches (Fig. 3 F). We further analyzed distant peri-glomerular regions, defined as areas located two to five glomerular radii from each glomerulus (Fig. S2 A). The pseudotime patterns in these regions were similar to those observed in peri-glomerular regions. TREM2^+^ macrophages were less abundant in the distant regions, suggesting that their accumulation was spatially associated with intra-glomerular sclerosis.

Clinically, the proportion of Damaged_PTCs in the peri-glomerular regions was inversely correlated with estimated glomerular filtration rate (eGFR) (Fig. 3 G). The proportions of FAP^+^ fibroblasts and TREM2^+^ macrophages within glomerular regions were positively correlated with the percentage of crescentic glomeruli at clinical diagnosis, consistent with their enrichment in injured glomerular tissue (Fig. 3 G). The correlations for all analyzed parameters are shown in Fig. S2 B. Overall, these data suggest that peri-glomerular PTC injury and immune cell accumulation emerge early, even around glomeruli without overt remodeling. Fibrogenic populations, including TREM2^+^ macrophages and FAP^+^ fibroblasts, accumulate in more advanced glomerular lesions. This pattern supports a model in which early PTC damage may help establish pathogenic immune cell niches that contribute to subsequent progression of glomerular injury.

### Damaged epithelial niches act as hubs for immune cell accumulation

**Figure 4.**
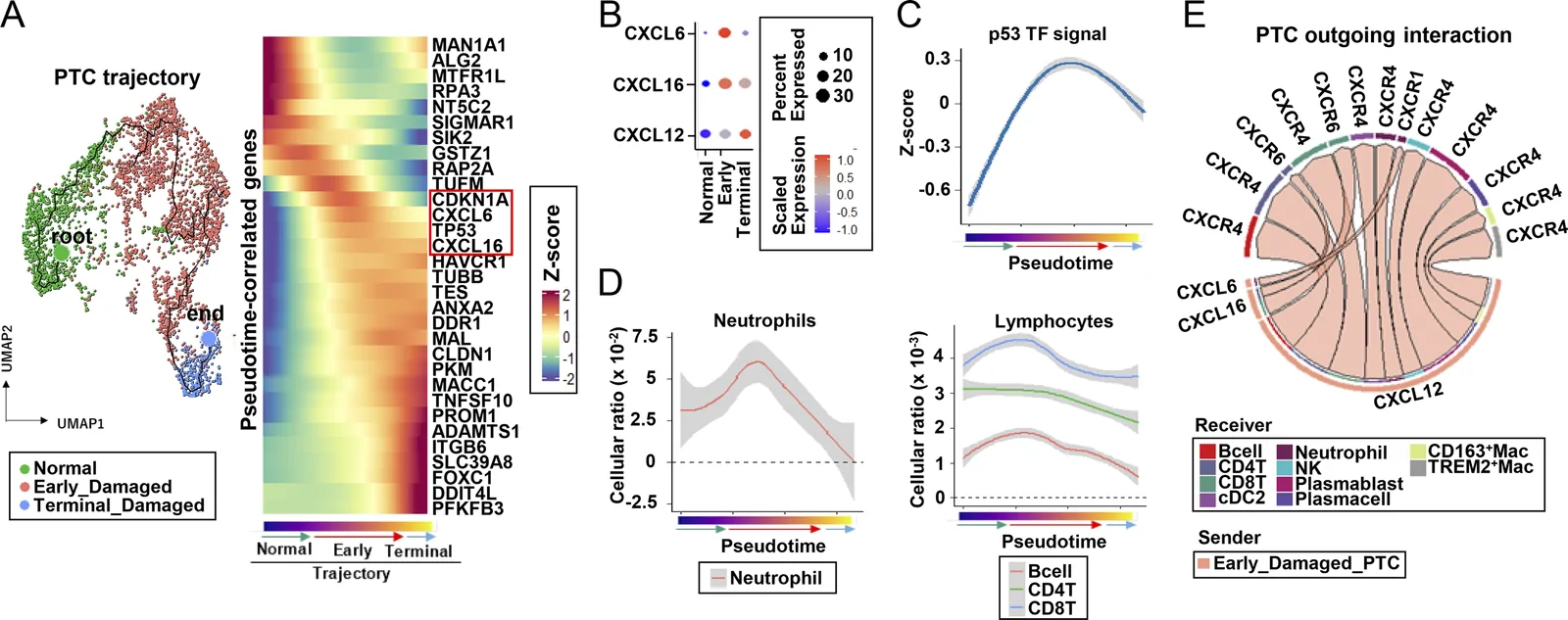
**Injured PTCs develop chemokine programs associated with local immune cell recruitment. (A)** Pseudotime trajectory analysis of PTCs showing inferred cellular state transitions associated with tubular injury. Black lines indicate trajectories originating from the Normal_PTC subset. Heatmap shows dynamic changes in representative genes across pseudotime. **(B)** Balloon plot showing the expression of chemokine genes across PTC injury lineages. Balloon color indicates the average of the scaled expression of each gene, and balloon size indicates the proportion of cells expressing the indicated genes. **(C)** Sequential changes in TP53 signaling score within 50 µm of each PTC along pseudotime. The x-axis represents pseudotime reflecting progression of PTC damage, and the y-axis indicates Z scores of TP53 signaling. Lines show the smoothed mean with 95% confidence intervals. **(D)** Immune cell accumulation within 50 µm of each PTC along pseudotime. The x-axis represents pseudotime reflecting progression of PTC damage, and the y-axis indicates neutrophil ratio and lymphocyte ratio. Lines show the smoothed mean with 95% confidence intervals. **(E)** Circos plot showing cell–cell interactions between Early_Damaged_PTCs (senders) and immune cell subsets (receivers). For each receiver population, spatial proximity integration and differential gene expression analysis were performed to identify enriched ligand–receptor pairs mediating intercellular signaling.

To further investigate the epithelial cell injury dynamics, we next performed a pseudotime trajectory analysis using Normal_PTCs as the starting point. PTCs were classified into three sequential states according to injury progression: Normal_PTC, Early_Damaged_PTC, and Terminal_Damaged_PTC (Fig. 4 A). During the transition from Normal_PTC to Early_Damaged_PTC, genes involved in cellular stress and damage sensing, including *CDKN1A* and *TP53*, were upregulated. Concomitantly, the expression of chemokine genes, such as *CXCL6* and *CXCL16*, increased in Early_Damaged_PTCs (Fig. 4 A). Notably, the upregulation of *CXCL6*, a gene encoding a potent neutrophil chemoattractant, was specific to the Early_Damaged_PTC state (Fig. 4 B). Consistent with these transcriptional changes, the activation of the p53 signaling pathway during this early phase coincided with an increase in the proportion of neutrophils, CD8^+^ T cells, and B cells in close proximity to the Damaged_PTC niches (Fig. 4, C and D). Cell–cell interaction analysis further revealed enriched CXCL signaling from Early_Damaged_PTCs, including CXCL12–CXCR4 signaling affecting diverse immune cell populations, as well as CXCL16–CXCR6 signaling to T cells and CXCL6–CXCR1 signaling to neutrophils (Fig. 4 E). Together, these data suggest that epithelial stress–associated transcriptional changes and immune cell accumulation occur in parallel during early tubular injury and that the Damaged_PTC niche acts as a hub for chemokine-mediated immune cell accumulation.

**Figure S3.**
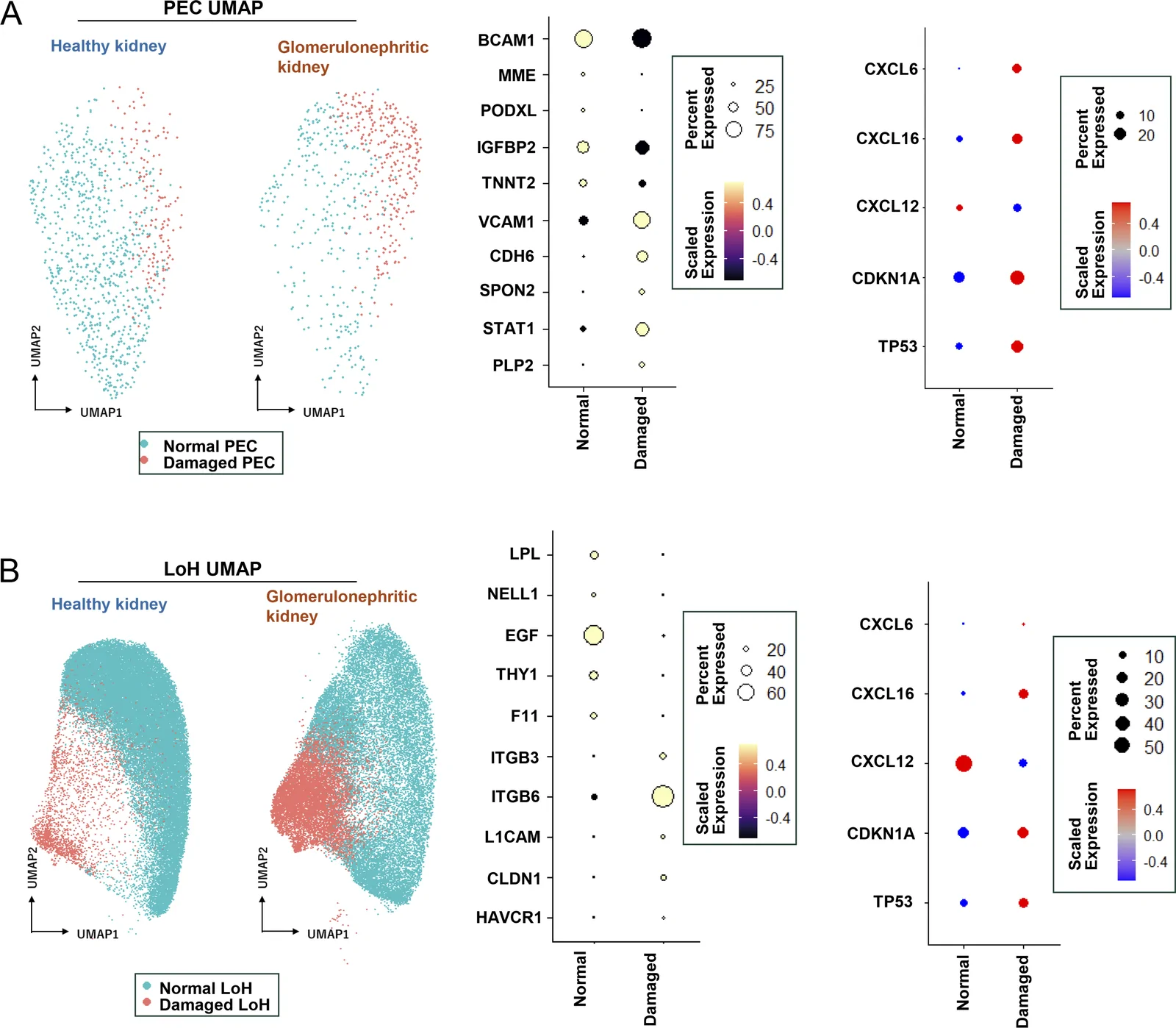
**Subset analysis of PEC and loop of Henle cell populations, related to Fig. 4. (A)** UMAPs of kidney PECs from healthy donors (left) and glomerulonephritis patients (right). Balloon plots showing representative marker gene expression and chemokine/cellular stress gene expression in each cell population. Balloon color indicates the average of the scaled expression of each gene, and balloon size indicates the proportion of cells expressing the indicated genes. **(B)** UMAPs of LoH from healthy donors (left) and glomerulonephritis patients (right). Balloon color and size are defined as in A.

We further assessed whether epithelial stress responses extend beyond PTCs to other epithelial lineages. PECs, which respond to local glomerular inflammation (Sultana et al., 2025), were segregated into normal and damaged populations (Fig. S3 A). Damaged_PECs, characterized by *VCAM1* expression, were significantly increased in the kidneys of patients and exhibited enhanced TP53 signaling and increased expression of *CXCL6* and *CXCL16*. Similar stress-associated transcriptional changes were observed in Damaged_LoH cells (Fig. S3 B), indicating that epithelial stress responses extend beyond PTCs to multiple epithelial segments.

### Damaged_PTC niches are enriched in distinct immune cell states, including FcγR-responsive neutrophils

**Figure 5.**
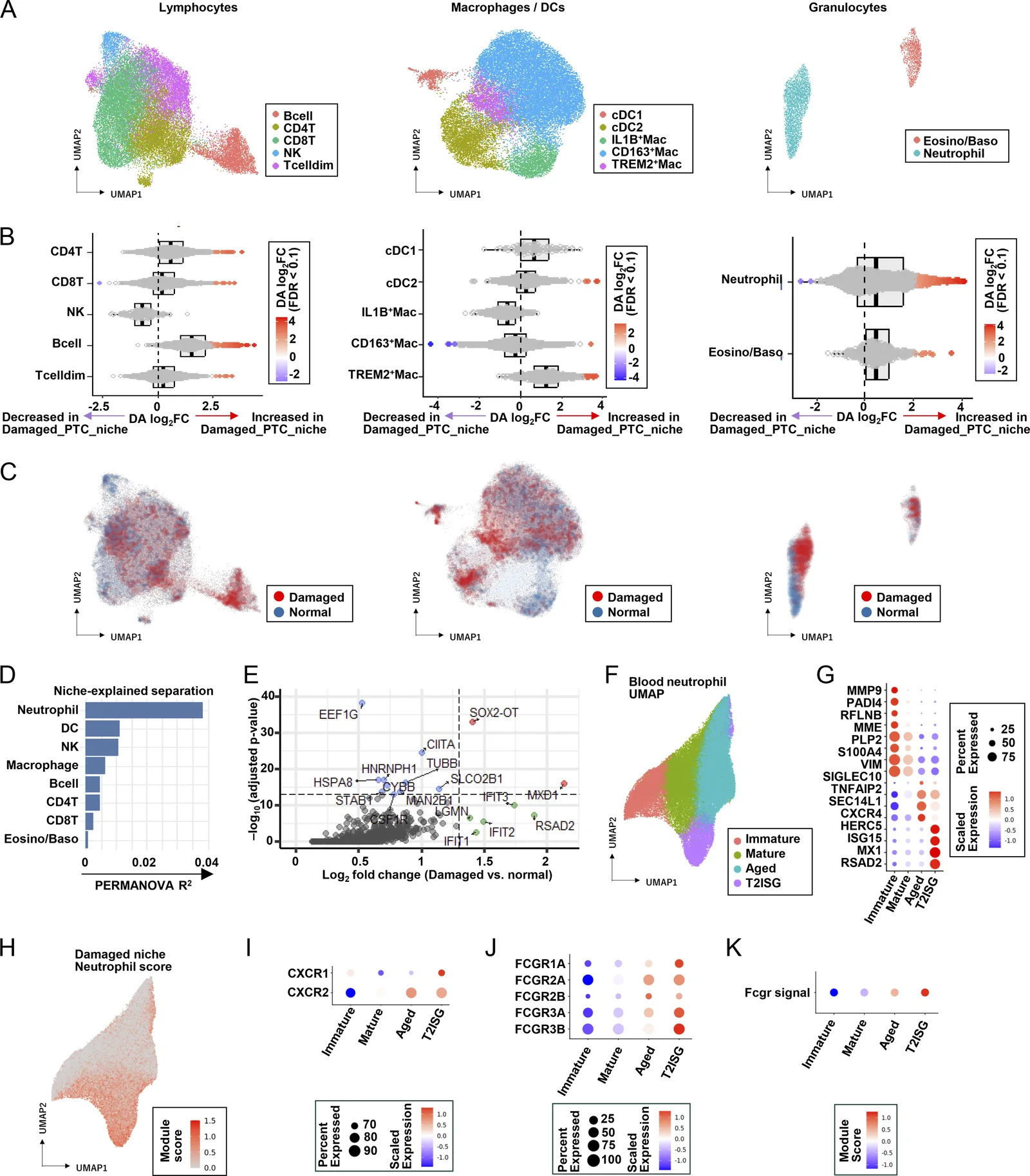
**Damaged_PTC niches harbor activated neutrophils with enriched type II interferon and FcγR signaling. (A)** UMAPs of kidney-infiltrating lymphocytes, macrophages/dendritic cells (DCs), and granulocytes. **(B)** Neighborhood graph of immune cell populations analyzed by Milo differential abundance testing. Nodes represent neighborhoods within each cell population. Colors indicate the log_2_-fold difference between Damaged_PTC and Normal_PTC niches for significantly differentially abundant neighborhoods (FDR < 0.10). Neighborhoods enriched in the Damaged_PTC niche are shown in red, and neighborhoods enriched in the Normal_PTC niche are shown in blue. **(C)** Density plots showing the distribution of Damaged_PTC-associated cells (red) and Normal_PTC-associated cells (blue) within each immune cell population. **(D)** Bar plot showing niche-associated UMAP-cluster variation in gene expression across immune cell types using PERMANOVA R^2^, indicating the proportion of transcriptional variance explained by niche localization. **(E)** Volcano plot showing DEGs in neutrophils localized to the Damaged_PTC niche compared with neutrophils from other niches. Genes with log_2_ fold change (log_2_FC) >1.3 and −log_10_(adjusted P value) >13 are highlighted and labeled. **(F)** UMAP of peripheral blood neutrophils from eight patients with ANCA-associated glomerulonephritis. **(G)** Balloon plots showing representative marker gene expression across peripheral neutrophil subsets. Balloon color indicates the average of the scaled expression of each gene, and balloon size indicates the proportion of cells expressing the indicated genes. **(H)** Module scoring of peripheral blood neutrophils using gene signatures derived from Damaged_PTC niche–associated kidney neutrophils. Cells are colored by scaled module score. **(I)** Balloon plot showing the expression of chemokine receptor genes across neutrophil subsets. **(J)** Balloon plots showing the expression of FcγR genes across neutrophil subsets. **(K)** Module scores of FcγR signaling pathways in neutrophils. Module scores were calculated based on scaled average gene expression, and the percentage of cells represents the proportion with a module score >0.

Next, we examined whether immune cells exhibited distinct activation states within Damaged_PTC niches. Integrated UMAPs of immune cells are shown in Fig. 5 A. We conducted differential abundance analysis using Milo, a cluster-free and unbiased analysis that identifies differences between cell populations by assigning cells to partially overlapping neighborhoods on a k-nearest-neighbor graph (Dann et al., 2022). As expected, neighborhoods significantly enriched in the Damaged_PTC niche (false discovery rate [FDR] < 0.10) were detected across immune cell populations, including CD4^+^ T cells, CD8^+^ T cells, B cells, cDC2, TREM2^+^ macrophages, and granulocytes (Fig. 5 B). Among these populations, neutrophils showed a clear separation between Damaged_PTC-associated (red) and Normal_PTC-associated (blue) cells in UMAPs (Fig. 5 C). Permutational multivariate ANOVA (PERMANOVA) analysis, which is a method for quantifying niche-associated transcriptional variations, confirmed that neutrophils exhibited the most pronounced niche-associated transcriptional divergence (Fig. 5 D). DEG analysis further revealed that type II interferon–stimulated genes (T2ISGs), such as *CIITA*, *CYBB*, and *IFIT3*, were enriched in Damaged_PTC niche–associated neutrophils (Fig. 5 E).

In peripheral blood, T2ISG-enriched neutrophils have been described as an MPO-ANCA–responsive population with enhanced ROS production and NET formation via FcγR signaling (Nishide et al., 2025). We next compared Damaged_PTC niche–associated neutrophils with peripheral blood neutrophils obtained from eight patients using single-cell RNA sequencing (scRNA-seq). Peripheral blood neutrophils were classified into four subsets based on their gene expression profiles: immature, mature, aged, and T2ISG-enriched neutrophils (Fig. 5 F). Representative marker gene expression patterns for each cluster are shown in Fig. 5 G. Module scoring analysis demonstrated that neutrophils within the Damaged_PTC niches closely resembled the peripheral blood T2ISG neutrophil subset (Fig. 5 H). These neutrophils expressed high levels of the CXCL6 receptors *CXCR1* and *CXCR2* (Fig. 5 I), along with increased expression of FcγRs (Fig. 5 J) and enhanced downstream FcγR signaling activity (Fig. 5 K). Together, these data indicate that neutrophils within the Damaged_PTC niche represent a distinct population and share features with circulating neutrophils characterized by increased autoantibody sensitivity.

### Development of a glomerulonephritis mouse model using pathogenic anti-MPO monoclonal antibodies

**Figure S4.**
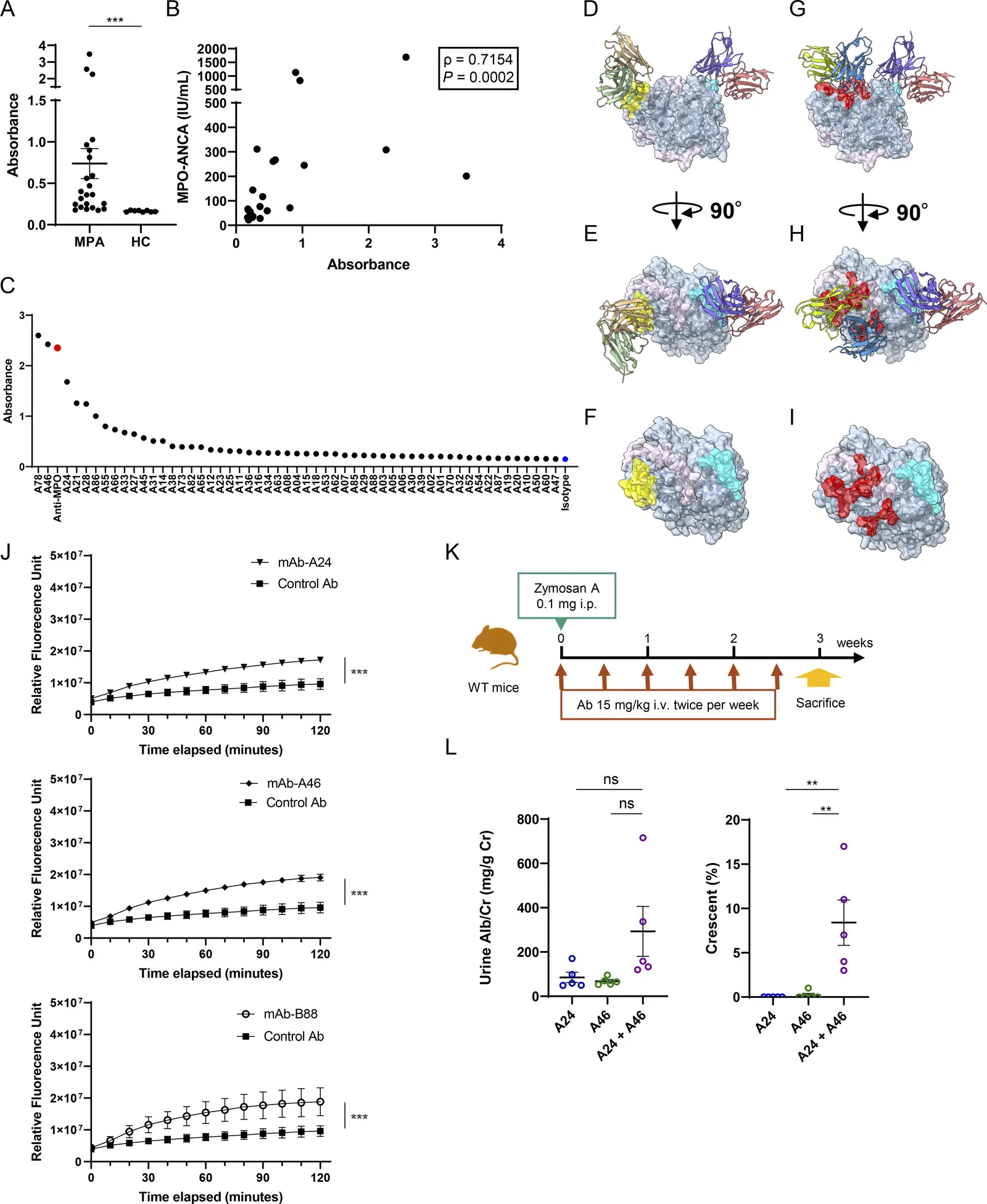
**Characterization and functional assessment of anti-MPO antibodies, related to Fig. 6. (A)** Sera from patients with MPO-AAV (MPA; *n* = 23) and healthy controls (HC; *n* = 8) were tested for IgG binding to immobilized full-length human MPO protein. The y-axis indicates ELISA OD values at 405 nm. Each dot represents an individual sample. Data are shown as mean ± SEM; ***P < 0.001; statistical significance was assessed using the Mann–Whitney U test. **(B)** Correlation between clinically determined MPO-ANCA titers and ELISA OD values. Spearman’s rank correlation coefficient (ρ) and P value are shown. **(C)** Purified mouse anti-MPO antibodies were tested for IgG binding to immobilized mouse MPO protein. The y-axis indicates ELISA OD values at 405 nm. The red dot indicates a commercially available anti-MPO antibody (Hycult Biotech) used as a positive control, and the blue dot indicates an isotype-matched control antibody used as a negative control. **(D–F)** Footprints of mAb-A24 (yellow) and mAb-A46 (cyan) on recombinant mouse MPO. **(G–I)** Footprints of mAb-B88 (red) and mAb-A46 (cyan) on MPO. Panels D and G show the complete ternary complexes; E and H present 90° rotated views; and F and I display MPO alone, with the contacted surface patches highlighted. MPO heavy and light chains are shown as semitransparent blue and pink surfaces, respectively. Antibody variable domains are depicted as ribbons in the same colors as in Fig. 6 C. Epitope residues (MPO atoms within 4 Å of any antibody atom) are shown as opaque surfaces in the indicated colors. **(J)** Bone marrow–derived neutrophils from wild-type mice were stimulated with either control anti-KLH antibody or a single anti-MPO monoclonal antibody. Extracellular ROS was measured fluorometrically every minute for 120 min. Representative traces show mean values from one experiment performed in triplicate. Data are presented as mean ± SEM; ***P < 0.001; statistical significance was assessed using one-way ANOVA with Tukey’s post hoc test. **(K)** Experimental workflow for antibody administration and disease induction. **(L)** Urinary albumin-to-creatinine ratios (left) and the percentage of glomeruli with crescents (right) were quantified in mice treated with a single antibody or a two-antibody combination. Data are presented as mean ± SEM; ns, not significant (P > 0.05), **P < 0.01; statistical significance was assessed by one-way ANOVA with Tukey’s post hoc test.

**Figure 6.**
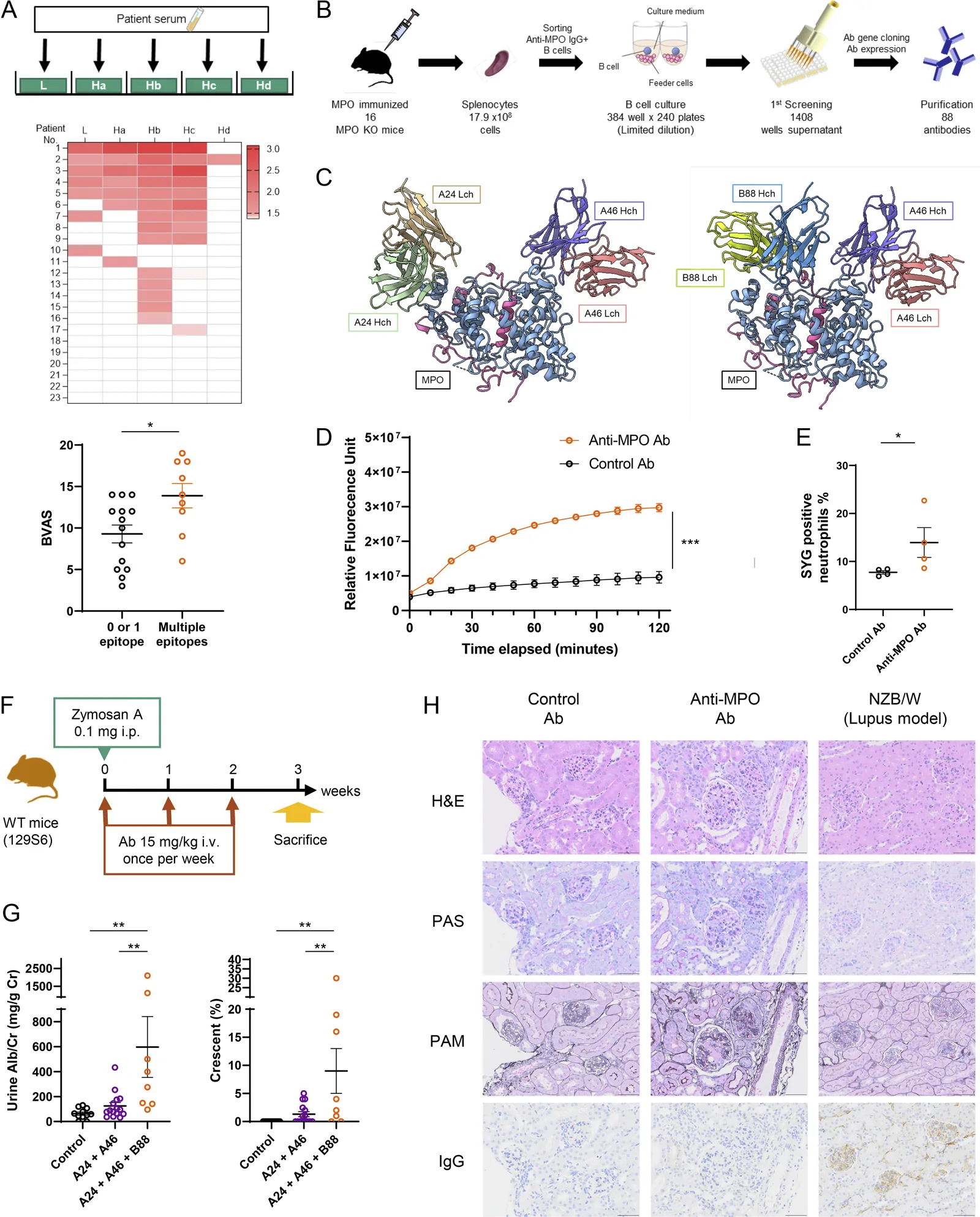
**Epitope-diverse anti-MPO antibodies enhance neutrophil activation and induce crescentic glomerulonephritis in mice. (A)** Mapping of MPO epitopes using truncated MPO fragments by ELISA. Sera from patients with MPO-AAV (*n* = 23) were tested for IgG binding to immobilized MPO fragments (L, Ha, Hb, Hc, and Hd). Heatmap shows OD values at 405 nm for each fragment across patients. Dot plot shows BVAS in patients with multi-epitope–reactive (*n* = 9) or single-epitope–reactive (*n* = 14) MPO-ANCA. Data are shown as mean ± SEM; *P < 0.05; statistical significance was assessed using the Mann–Whitney U test. **(B)** Experimental workflow for the generation of mouse anti-MPO monoclonal antibodies. MPO-deficient mice were immunized with recombinant mouse MPO, and splenic MPO-specific IgG^+^ B cells were isolated and screened for MPO binding by ELISA. Variable regions from selected clones were cloned into expression vectors containing mouse IgG2a constant regions. **(C)** Cryo-electron microscopy structures of MPO–antibody complexes. Recombinant mouse MPO in complex with antibodies mAb-A24 and mAb-A46 (left), and with mAb-A46 and mAb-B88 (right). Ribbon representations are color-coded as follows: MPO light chain (pink) and heavy chain (blue); mAb-A24 light chain (tan) and heavy chain (green); mAb-A46 light chain (red) and heavy chain (purple); mAb-B88 light chain (yellow) and heavy chain (cyan). **(D)** Effects of anti-MPO monoclonal antibodies on neutrophil ROS production. Bone marrow–derived neutrophils from wild-type mice were stimulated with control antibodies or combinations of anti-MPO monoclonal antibodies. Extracellular ROS was measured fluorometrically every minute for 120 min. Representative traces show mean values from one experiment performed in triplicate. Data are presented as mean ± SEM; ***P < 0.001; statistical significance was assessed using one-way ANOVA with Tukey’s post hoc test. **(E)** Effects of anti-MPO monoclonal antibodies on NET formation *in vivo*. Wild-type mice were primed with intraperitoneal TNF-α, followed by injection of control antibodies or anti-MPO monoclonal antibody combinations. Peritoneal exudate neutrophils were analyzed 2 h later. NET formation was quantified as Ly6G^+^ SYTOX Green^+^ cells. Data are shown as mean ± SEM; *P < 0.05; statistical significance was determined using the Mann–Whitney U test. **(F)** Experimental workflow for induction of crescentic glomerulonephritis in mice using combinations of anti-MPO monoclonal antibodies. **(G)** Urinary albumin-to-creatinine ratios (left) and the percentage of crescentic glomeruli (right) in mice treated with control antibodies, a two-antibody combination, or a three-antibody combination. Data are presented as mean ± SEM; **P < 0.01; statistical significance was assessed using one-way ANOVA with Tukey’s post hoc test. **(H)** Representative kidney histology from mice treated with control antibodies or anti-MPO monoclonal antibodies. Kidney tissue from an NZB/W F1 lupus-prone mouse is shown as a positive control for immune complex–mediated nephritis. Sections were stained with H&E, periodic acid–Schiff (PAS), periodic acid–methenamine silver (PAM), and by direct immunofluorescence for IgG. Scale bar, 50 μm.

Spatial transcriptomic analysis of patient kidneys implicated early neutrophil activation characterized by enhanced FcγR signaling, linked to epithelial stress responses. Given that FcγR engagement is critically influenced by the spatial density and epitope diversity of antigen-bound IgG (Gallo et al., 2010; Patel et al., 2019), we next examined the epitope specificity of MPO-ANCA in patient sera by ELISA. Full-length MPO and MPO fragments (Erdbrugger et al., 2006) were pre-coated, and sera from 23 newly diagnosed, treatment-naïve patients with MPO-ANCA–associated vasculitis (AAV) were used to quantify antibody binding. Detailed clinical information is provided in Table S3. The binding intensity of patient sera to full-length MPO was significantly stronger than that of healthy sera (Fig. S4 A), and the binding intensity correlated with serum MPO-ANCA titers at clinical diagnosis (Fig. S4 B). Epitope mapping revealed that 9 of the 23 patients recognized multiple MPO fragments, with binding to the heavy-chain Hb and/or Hc regions observed in all cases (Fig. 6 A). These patients exhibited significantly higher Birmingham Vasculitis Activity Scores (BVAS) (Fig. 6 A), suggesting an association between multi-epitope recognition and disease activity.

To validate these observations experimentally, we established a mouse model of MPO-ANCA–associated glomerulonephritis using pathogenic anti-MPO monoclonal antibodies. MPO-deficient mice were immunized with recombinant MPO. In the first screening campaign, MPO-binding IgG^+^ B cells were isolated from the spleens of 20 immunized mice, and candidate antibodies were screened for binding to recombinant MPO by ELISA (Fig. S4 C). Among the antibodies with strong MPO-binding activity, mAb-A24 and mAb-A46 were successfully produced and purified. In the second campaign, mAb-B88 was purified at scale after initial functional testing confirmed that it additively enhanced neutrophil activation when combined with mAb-A24 and mAb-A46, as described below. Cryo-electron microscopy (cryo-EM) epitope mapping showed that mAb-A24 bound to the Hc helix–loop–helix region (P583–E599), mAb-A46 bound to the Hb loop (N487–R498), and mAb-B88 recognized two regions within Hc (R210–E221 and R533–M693) (Fig. 6 C and Fig. S4, D–I). The aa residues on MPO identified as epitopes for each monoclonal antibody are listed in Table S4.

Functionally, each antibody induced ROS production in mouse neutrophils (Fig. S4 J). When administered in combination at equivalent total antibody concentrations, ROS production was markedly enhanced relative to single-antibody stimulation (Fig. 6 D), and NET formation was induced in peritoneal neutrophils (Fig. 6 E). In vivo, the administration of mAb-A24 or mAb-A46 alone did not induce overt glomerulonephritis, whereas their combined administration triggered renal injury (Fig. S4, K and L). The addition of mAb-B88 resulted in a more severe disease with fewer injections, characterized by urinary abnormalities and prominent cellular crescent formation (Fig. 6, F and G). Histological analysis revealed crescent formation and glomerular fibrosis with minimal IgG deposition, reproducing the pauci-immune pattern characteristic of human ANCA-associated glomerulonephritis (Fig. 6 H). These results demonstrate that combinations of anti-MPO monoclonal antibodies recognizing distinct epitopes enhance neutrophil activation and induce glomerulonephritis with histopathological features that closely resemble those of human disease.

### Spatial transcriptomics of the anti-MPO antibody–induced mouse model recapitulates human disease-associated renal niches

**Figure 7.**
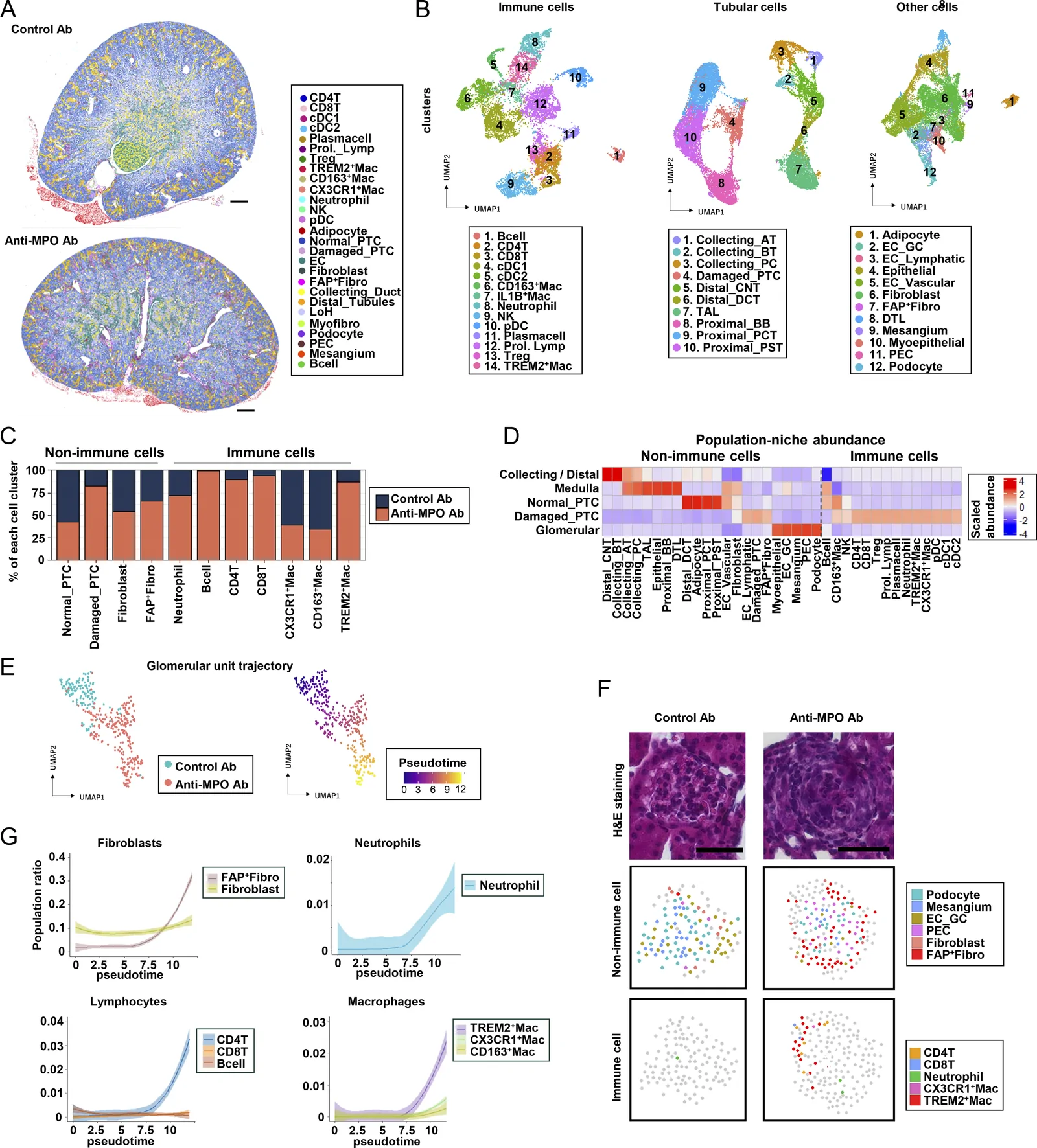
**Spatial transcriptomics of an anti-MPO antibody–induced mouse model recapitulates key features of human ANCA-associated glomerulonephritis. (A)** Representative spatial cell type maps of kidney sections from a control antibody–treated mouse and an anti-MPO antibody–treated mouse, generated by Xenium spatial transcriptomics. Cell segmentation and annotation were performed as in Fig. 1 B. Cells are colored by annotated cell type (abbreviations shown; see Fig. S5 A for full definitions). **(B)** UMAPs of immune cells, tubular epithelial cells, and other stromal cell populations from integrated mouse kidney datasets. **(C)** Stacked bar plots showing the proportions of the indicated cell populations among all kidney cells in a control antibody–treated mouse and an anti-MPO antibody–treated mouse. **(D)** Heatmap showing the enrichment scores for each cell subpopulation across five proximity-informed niches in the mouse model. Niche extraction and annotation were performed as in Fig. 2 B. **(E)** UMAP-based clustering (left) and pseudotime trajectory analysis (right) of glomerular niches extracted from mouse kidney sections. Glomeruli were classified into two clusters based on pseudobulk gene expression profiles. **(F)** Representative histopathological features and corresponding spatial transcriptomic cell type maps for glomerular regions from a control antibody–treated mouse and an anti-MPO antibody–treated mouse. Cells are colored by cell type annotation. Scale bar, 50 μm. **(G)** Sequential changes in the indicated cell populations across pseudotime in regions within glomerular niches (intra-glomerular). The x-axis represents pseudotime reflecting disease progression, and the y-axis indicates the relative abundance of each cell subset. Lines show the smoothed mean with 95% confidence intervals.

**Figure S5.**
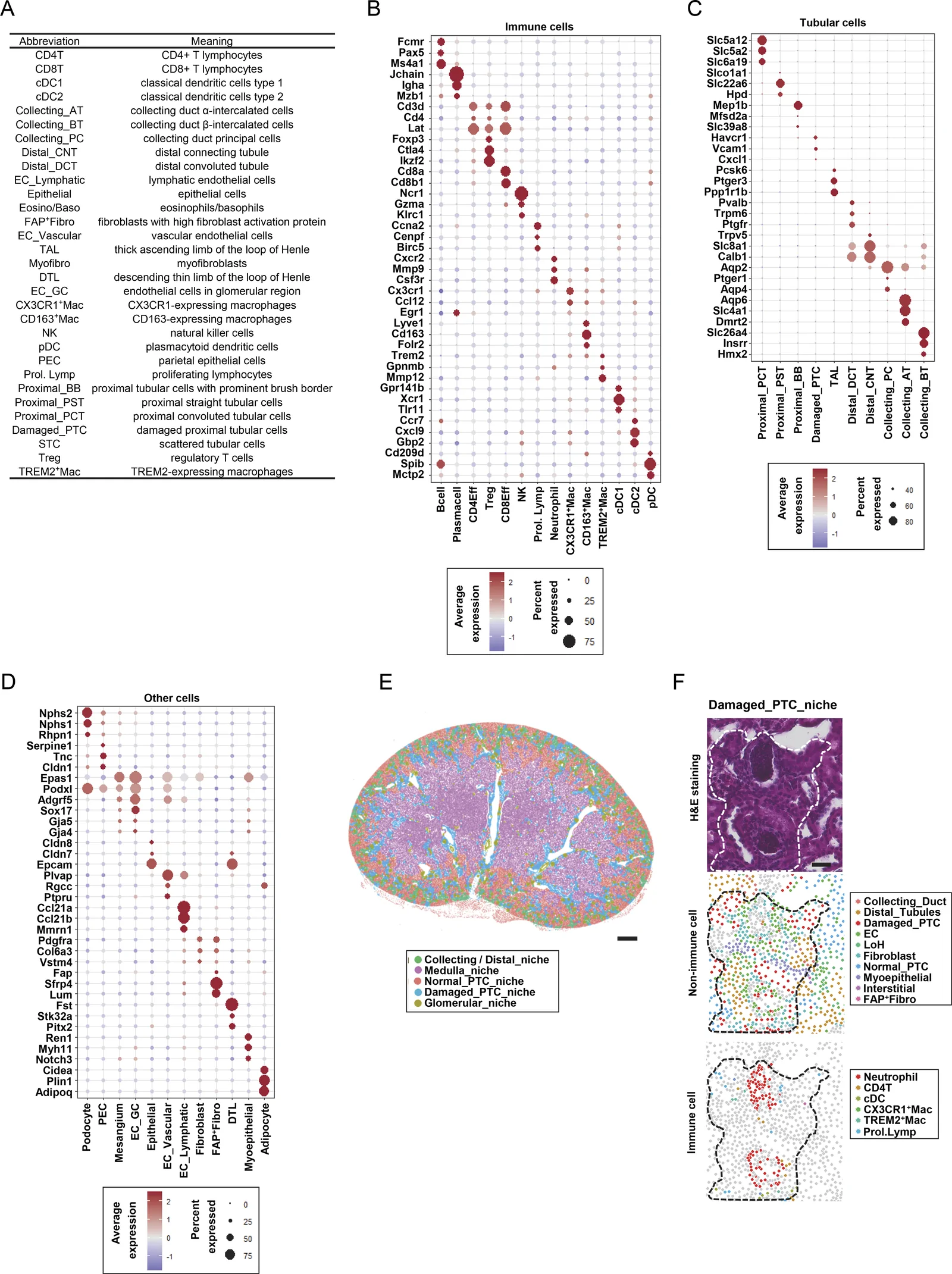
**Cell annotation and spatial niche organization in anti-MPO antibody–treated mouse kidneys, related to Fig. 7. (A)** Abbreviations and definitions of mouse renal cell clusters. **(B)** Balloon plot showing highly expressed genes in immune cell populations within mouse kidney tissues. Balloon color indicates the average of the scaled expression of the indicated genes. Balloon size indicates the percentage of cells expressing the indicated genes. **(C)** Balloon plot showing highly expressed genes in tubular cell populations within mouse kidney tissues. **(D)** Balloon plot showing highly expressed genes in other cell populations within mouse kidney tissues. **(E)** Representative spatial transcriptomics–based niche maps from anti-MPO antibody–treated mouse kidneys. Five cellular niches were identified based on spatial proximity analysis of transcriptomic data. Scale bar, 500 μm. **(F)** Representative regions of mouse Damaged_PTC niches shown by H&E staining and spatial cell type annotation maps of nonimmune and immune cell populations. The niche region is indicated by a white dashed line. Scale bar, 50 μm.

Next, we performed spatial transcriptomic analysis of kidneys from control antibody–treated mice and from an anti-MPO antibody–induced glomerulonephritis mouse model. Whole-kidney cell type maps from a control antibody–treated mouse and an anti-MPO antibody–induced glomerulonephritis mouse are shown in Fig. 7 A. The cell type abbreviations used for annotation are listed in Fig. S5 A, and marker gene expression of immune cells, tubular cells, and other renal cells is shown in Fig. S5, B–D, respectively. The integrated UMAPs are shown in Fig. 7 B. Similar to the human disease, mice treated with anti-MPO antibodies showed enrichment of Damaged_PTCs and FAP^+^ fibroblasts, with increased immune cell infiltration including neutrophils, B cells, CD4^+^ T cells, CD8^+^ T cells, and TREM2^+^ macrophages (Fig. 7 C). Niche analysis identified five major niches, including collecting duct/distal tubule, medullary, normal PTC, Damaged_PTC, and glomerular niches (Fig. S5 E and Fig. 7 D). A representative cell map of the Damaged_PTC niche is shown in Fig. S5 F. In mice, glomerular niches were classified into two distinct clusters, and pseudotime trajectory analysis revealed a continuous shift in gene expression along the glomerular injury axis (Fig. 7 E). Representative glomerular regions from each cluster are shown in Fig. 7 F, with the corresponding H&E-stained images and separate spatial maps of nonimmune and immune cell populations. Advanced glomeruli in the anti-MPO antibody–induced glomerulonephritis mouse model showed proliferative and crescentic changes with local accumulation of fibrogenic cell populations, including FAP^+^ fibroblasts and TREM2^+^ macrophages (Fig. 7 F). Glomerulus-level pseudotime analysis in the mouse model showed that CD4^+^ T cells and neutrophils accumulated within diseased glomeruli along the injury trajectory. At the sclerotic stage, FAP^+^ fibroblasts and TREM2^+^ macrophages markedly expanded within glomerular niches (Fig. 7 G). These features recapitulated key aspects of the glomerular changes observed in human ANCA-associated glomerulonephritis.

### One-armed anti-MPO antibodies suppress FcγR-dependent neutrophil activation and attenuate glomerular injury

**Figure 8.**
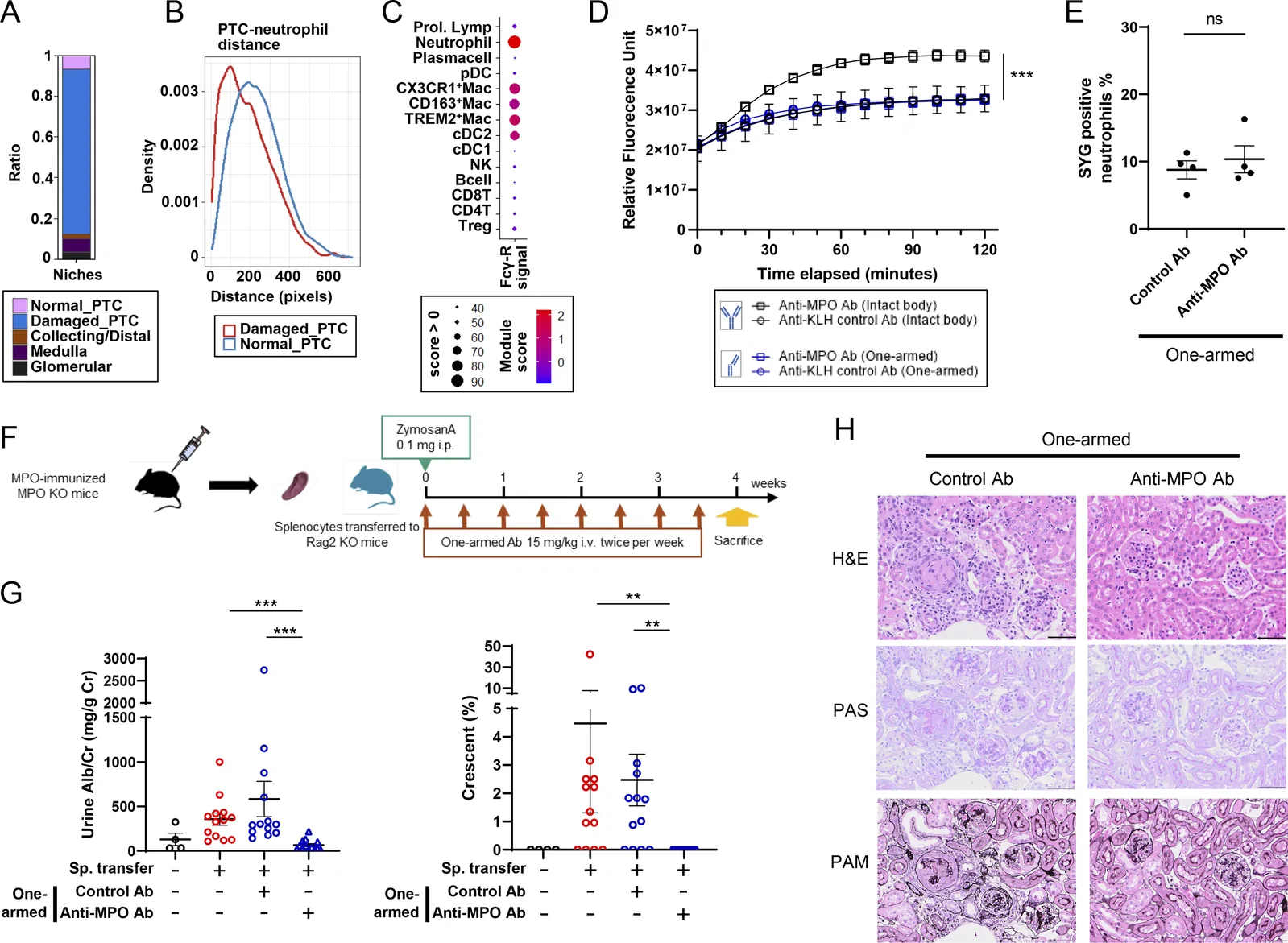
**One-armed anti-MPO antibodies suppress neutrophil activation and attenuate splenocyte transfer–induced MPO-ANCA glomerulonephritis. (A)** Stacked bar graph showing the spatial distribution of neutrophils across proximity-defined niches in the mouse model. **(B)** Histograms showing the shortest distance (pixels) from Normal_PTC and Damaged_PTC to the nearest neutrophil. **(C)** Module scoring analysis of FcγR signaling activity across immune cell subsets in the mouse model. Cells are colored according to their scaled module scores. **(D)** Effects of one-armed anti-MPO antibodies on neutrophil ROS production. Bone marrow–derived neutrophils from wild-type mice were stimulated with intact or one-armed control antibodies, or with intact or one-armed anti-MPO monoclonal antibodies. Extracellular ROS production was measured as in Fig. 6 D. Data are presented as mean ± SEM; ***P < 0.001; statistical significance was assessed by one-way ANOVA with Tukey’s post hoc test. **(E)** Effects of one-armed anti-MPO antibodies on NET formation. NETosis was induced and quantified as in Fig. 6 E. Data are presented as mean ± SEM; ns, not significant (P > 0.05); statistical significance was determined using a two-sided Mann–Whitney U test. **(F)** Experimental workflow for the passive-transfer AAV mouse model and therapeutic intervention. MPO-deficient mice were immunized with recombinant MPO, and splenocytes were transferred into Rag2-deficient recipient mice to induce glomerulonephritis. One-armed anti-MPO antibodies or control antibodies were administered intravenously (15 mg/kg, twice per week) from day 0 to week 3. **(G)** Therapeutic efficacy of one-armed anti-MPO antibodies in the passive-transfer AAV model. Urinary albumin-to-creatinine ratios (left) and percentages of glomeruli with crescents (right) were assessed at week 4. Data are presented as mean ± SEM; **P < 0.01; ***P < 0.001; statistical significance was determined by one-way ANOVA followed by Tukey’s multiple-comparisons test. **(H)** Representative renal histopathology from passive-transfer AAV mice treated with one-armed control antibodies or one-armed anti-MPO antibodies. Sections were stained with H&E, PAS, and PAM. Scale bar, 50 μm. PAS, periodic acid–Schiff; PAM, periodic acid–methenamine silver.

The Damaged_PTC niche in the mouse model recapitulated the immune-enriched spatial architecture observed in human kidneys. Notably, more than 80% of kidney-infiltrating neutrophils were localized to the Damaged_PTC niches (Fig. 8 A), and Damaged_PTCs were significantly more likely to be found in close proximity to neutrophils than Normal_PTCs (Fig. 8 B). Consistent with the findings in human kidneys, FcγR downstream signaling activity among renal immune cells was highest in neutrophils (Fig. 8 C).

Based on these observations, we tested whether selectively blocking FcγR-mediated neutrophil activation could ameliorate disease. We engineered one-armed anti-MPO antibodies that preserved antigen binding while minimizing FcγR engagement. One-armed variants of mAb-A24, mAb-A46, and mAb-B88 failed to induce neutrophil ROS production (Fig. 8 D) and reduced NET formation to levels comparable with those of the control antibodies (Fig. 8 E). In a passive-transfer AAV model (Fig. 8 F) (Xiao et al., 2002), treatment with one-armed antibodies significantly improved urinary abnormalities and attenuated glomerular pathology (Fig. 8 G), with marked reductions in cellular crescents and inflammatory infiltration (Fig. 8 H). These results indicate that one-armed antibodies targeting pathogenic MPO epitopes can selectively inhibit FcγR-dependent neutrophil activation while preserving antigen specificity.

In summary, the findings reveal the spatial organization of pathogenic events in autoimmune glomerulonephritis. Peri-glomerular PTC stress responses shape a chemokine-enriched niche that accumulates immune cells, including activated neutrophils characterized by T2ISG expression and enhanced FcγR signaling. Along the pseudotime trajectory, immune infiltration extends into the glomerulus, followed by the expansion of fibrogenic macrophages and fibroblast subsets. A pathogenic anti-MPO antibody–driven mouse model recapitulates these features, and selective blockade of FcγR-dependent neutrophil activation using one-armed antibodies attenuates glomerular injury in vivo.

## Discussion

Autoimmune glomerulonephritis does not result from random immune infiltration but is organized via spatially defined renal niches that coordinate immune, epithelial, and stromal activation (Lake et al., 2023; Melo Ferreira et al., 2021; Reck et al., 2025). By reconstructing glomerular niche states using pseudotime analysis, our study suggests that pathogenic events may emerge in peri-glomerular microenvironments before overt glomerular inflammation becomes apparent. Spatial maps revealed that stressed epithelial cells activate chemokine programs that recruit and retain neutrophils, T cells, and B cells, thereby establishing a permissive immune niche that precedes glomerular lesion progression.

PTC injury onset can be attributed to diverse insults, including ischemia, exposure to damage-associated molecular patterns, and release of inflammatory mediators by infiltrating immune cells (Tang et al., 2019). A key finding of this study was that p53 pathway activation in Early_Damaged_PTCs coincided with increased immune cell accumulation around injured tubules. This association suggests that epithelial stress may contribute actively to local inflammation rather than merely serve as a marker of tubular damage. Tubular p53 activation has been shown to promote inflammatory cytokine transcription through the SIRT1–p65/NF-κB axis (Fu et al., 2022), whereas PTC-specific deletion of p53 attenuates ischemia-induced acute kidney injury and the accompanying inflammatory responses (Ying et al., 2014). These findings support the possibility that p53 activation in Early_Damaged_PTCs contributes to a local cytokine and chemokine milieu that facilitates immune cell recruitment. The ligand–receptor interactions detected within Damaged_PTC niches are consistent with this model, suggesting functional cross talk between epithelial injury and immune cell localization in ANCA-associated glomerulonephritis.

More broadly, these epithelial–immune interactions place the pathogenic trigger of ANCA-associated glomerulonephritis within the biology of maladaptive tubular repair. Spatial transcriptomic studies of mouse acute kidney injury have identified fibro-inflammatory niches comprising failed-repair PTCs, fibroblasts, and infiltrating immune populations (Xuanyuan et al., 2025). Within this shared injury program, CXCL16 produced by myeloid cells may recruit or retain CXCR6^+^ T cells near damaged tubules. Experimental evidence indicates that this axis promotes renal T cell accumulation, PTC death, fibrosis, and loss of kidney function during maladaptive repair (Li et al., 2026). Thus, CXCL16–CXCR6-dependent T cell localization may represent a general mechanism of persistent tubular injury rather than a response specific to ANCA-associated glomerulonephritis. By contrast, CXCL6–CXCR1/2-dependent recruitment of FcγR-responsive neutrophils to Damaged_PTC niches may be more directly linked to the antibody-dependent pathogenesis of AAV. Together, these findings suggest that ANCA-associated glomerulonephritis shares features of a conserved maladaptive repair program, while its disease-specific inflammatory character may be shaped by antibody-driven neutrophil recruitment and activation.

Neutrophils within Damaged_PTC niches represent a tissue-damaging subset characterized by T2ISGs and enhanced FcγR signaling (Nishide et al., 2025). Although the temporal hierarchy between neutrophil activation and epithelial stress cannot be fully determined, epithelial stress and neutrophil influx may initially arise in parallel and subsequently reinforce each other in a feed-forward amplification loop that accelerates tissue injury. In clinical practice, conventional clinical markers are poorly suited for detecting early fibrogenic shifts (Kidney Disease: Improving Global Outcomes, 2024; Levin and Stevens, 2011). In our study, peri-glomerular Damaged_PTC signatures were correlated with reduced eGFR at shorter disease durations, suggesting that they may serve as indicators of prefibrotic states that precede tissue remodeling. However, these patient-level correlations should be interpreted cautiously because of spatial heterogeneity within biopsy samples, which may be affected by compositional changes in lesion-associated populations. Validation in larger cohorts will be required to confirm these associations.

Along the trajectory of disease progression, we identified two fibrogenic cell populations, TREM2^+^ macrophages and FAP^+^ fibroblasts, that expand within advanced glomerular niches. TREM2^+^ macrophages share transcriptional features with SAMs described in liver fibrosis, whereas FAP^+^ fibroblasts adopt programs resembling mCAFs, highlighting conserved profibrotic transcriptional states across tissues. Spatially, these populations accumulated predominantly in the crescentic glomeruli, and their abundance correlated with the extent of crescent formation, indicating that fibrogenic remodeling emerged as a downstream consequence of earlier immune–epithelial interactions.

To mechanistically validate this neutrophil–epithelial axis, we developed monoclonal anti-MPO antibodies that directly activated neutrophils and established an antibody-induced model of glomerulonephritis. Previous attempts to induce glomerulonephritis in wild-type mice using single monoclonal anti-MPO antibodies have been limited, suggesting that antibody binding alone is insufficient to trigger the disease (Huang et al., 2023a; Shochet et al., 2020). In our study, the enhanced disease induction by the A24/A46/B88 combination may reflect an antibody-binding geometry that favors FcγR clustering. Although mAb-A46 and mAb-B88 bound overlapping regions on the MPO heavy-chain surface, cryo-EM analysis showed that they recognized distinct residues and approached MPO from different angles. We therefore speculate that these differences alter the orientation of exposed Fc domains and thereby facilitate productive FcγR engagement, consistent with previous evidence that Fc receptor activation depends on both Fc valency and multivalent Fc organization (Klaassen et al., 1988; Ortiz et al., 2016). Supporting this model, one-armed anti-MPO antibodies that preserved antigen binding while minimizing FcγR engagement failed to activate neutrophils and effectively attenuated glomerular injury, highlighting FcγR-dependent neutrophil activation as a tractable therapeutic target.

This study has several limitations. First, human biopsy data represent cross-sectional snapshots, and pseudotime analyses provide computational inferences rather than direct temporal measurements. Longitudinal spatial sampling is required to resolve causal relationships definitively. Second, the antibody-induced model relies on strain-specific innate immune responsiveness, with severe disease development in 129S6 mice that exhibit heightened neutrophil reactivity (Jones et al., 2020; Mekada and Yoshiki, 2021). Finally, the three one-armed antibodies used in this study should not be interpreted as covering all pathogenic epitopes within the polyclonal anti-MPO repertoire. Rather, occupancy of pathogenicity-enriched Hb/Hc regions may interfere with antibody-binding geometries that favor FcγR-dependent neutrophil activation, even when other anti-MPO antibodies remain able to bind MPO. Further epitope-resolved analyses will be required to determine how the geometric arrangement of antibody-binding sites on MPO influences FcγR-dependent neutrophil activation.

In conclusion, this study integrated spatial pathology, mechanistic modeling, and therapeutic intervention to demonstrate that peri-glomerular proximal tubules act as central hubs connecting neutrophil activation to progressive immune and stromal remodeling. The interruption of the earliest events of neutrophil activation may represent an effective strategy for preventing fibrogenic progression. More broadly, cross-disease spatial analyses may reveal conserved epithelial stress circuits underlying immune-mediated tissue injury and identify opportunities for early mechanism-based therapeutic interventions.

## Materials and methods

### Study participants and sample preparation

All clinical samples were collected from study participants who provided informed consent, in accordance with the Declaration of Helsinki. Serum samples were collected with approval from the ethics review board of the Graduate School of Medicine, The University of Osaka, Japan (No. 24135). Consent was obtained to publish information such as age, sex, the name of the medical center, and the diagnosis. The sex of each study participant was determined based on self-report. Study participants received no compensation.

Eight patients with MPA (five females; median age, 74.5 years) and four healthy donors (two females; median age, 68 years) were recruited for spatial transcriptome analysis of kidney tissue. Stored sera from 23 patients (12 females; median age, 73 years) and eight healthy donors (four females; median age, 68.1 years) were used for the MPO epitope determination assay. Kidney tissue samples from MPA patients were biopsied for diagnosis. Kidney tissue samples from healthy donors were obtained from donor kidneys at the time of kidney transplantation. The tissues were fixed in 10% neutral buffered formalin, and then embedded in paraffin for further analysis. For serum samples, whole blood (3.5 ml) was drawn into Vacutainer SST II tubes (Cat. No.: 365920; BD Diagnostics) and centrifuged for 10 min at 1,200 × *g*. The supernatant, designated as serum, was then stored at −80°C. MPA diagnosis in patients with AAV conformed to the 2022 American College of Rheumatology/European Alliance of Associations for Rheumatology classification criteria (Suppiah et al., 2022a; Suppiah et al., 2022b) and was confirmed by at least two rheumatologists. The BVAS 2008 version 3 was used to assess MPA disease activity. All involved patients were newly diagnosed, had not undergone any immunosuppressive therapy, and were admitted to the University of Osaka Hospital. Comprehensive assessments were conducted to exclude other potential causes resembling vasculitis, such as infectious diseases and neoplastic lesions, before applying the criteria.

### Mice

C57BL/6J wild-type mice were purchased from CLEA Japan, Inc. 129S6 wild-type mice (129S6/SvEvTac) were purchased from Taconic Biosciences. MPO-deficient mice (B6.129X1-*Mpo*^*tm1Lus*^/J) were purchased from Jackson Laboratories. Rag2-deficient recipient mice were provided by RIKEN BRC through the National BioResource Project, MEXT. For mouse analysis, littermate controls were used. All the mice used in this study were housed in a pathogen-free facility. All the protocols were approved by the Animal Research Committee of the Immunology Frontier Research Center (The University of Osaka, Japan).

### Xenium spatial transcriptomics assay

For spatial analysis, 5-µm-thick kidney sections from MPA (*n* = 8) and healthy donors (*n* = 4) were placed on Xenium slides. In situ gene expression data, cell-segmentation staining, and post-Xenium H&E staining were generated using the 10x Genomics Xenium In Situ 5k-plex platform according to the manufacturer’s instructions (10x Genomics), including fixation and permeabilization as per the vendor protocol.

### Data processing of spatial transcriptomic data

The Seurat R package (version 4.2.0) was used for data quality control, scaling, transformation, clustering, dimensionality reduction, differential expression analysis, and visualization. Low-quality cells with nCount_Xenium values <10 were removed, and a total of 410,222 cells were retained for downstream analysis. Data from all samples were integrated using Seurat’s CCA-based integration, with the FindVariableFeatures (method = “vst”, nfeatures = 3,000), the FindIntegrationAnchors (dims = 1:25), and the IntegrateData function run under set.seed(1234). The RunUMAP function was performed on the first 25 principal components (PCs). A shared nearest neighbor graph was constructed on the same 25 PCs using the FindNeighbors function, followed by Louvain clustering using the FindClusters function. UMAP embeddings were visualized using the DimPlot function. The Xenium assay was set as the default assay (DefaultAssay = “Xenium”), and data were normalized (NormalizeData with method = “LogNormalize”) and scaled (ScaleData) prior to downstream analyses. Cluster-level mean expression was computed using the AverageExpression function with groupings by clusters.

### Niche analysis

Cellular niches were identified using the centroid of each cell. For every cell, we enumerated neighbors within a 50 µm radius and computed a cell type composition vector to form a cell population matrix, with sensitivity analyses at radii of 30 and 100 µm to verify robustness. We then ran UMAP on this matrix and performed Louvain clustering after constructing a nearest-neighbor graph on the first 11 PCs (FindNeighbors followed by FindClusters). The composition of each neighborhood was visualized using heatmaps. The mean nearest-neighbor distance between specified cell types was computed using the FNN package, and the get.knnx function was used to efficiently compute distances between two specified populations.

### Glomerular unit trajectory analysis

To analyze the progression of individual glomeruli toward crescent formation, single glomerular units were identified. After subsetting glomerular niches, we used xy coordinates with the dbscan (version 1.2.2). We used the hdbscan function to spatially segment glomerular niches; clusters containing ≥20 cells were defined as glomerular units. Following visual inspection, if multiple glomeruli were identified within the same cluster, we performed additional subclustering to resolve them and confirmed that each resulting cluster corresponded to a single glomerulus. For each unit, mean expression was computed using the AverageExpression function, and the top 300 most variable genes were used to construct a UMAP with the umap function (n_neighbors = 20, min_dist = 0.2, metric = “cosine”). Units were clustered using k-means (centers = 6, nstart = 50), and pseudotime was estimated using Slingshot. Slingshot trajectories were fitted on UMAP coordinates with start.clus set to the cluster corresponding to the normal-appearing glomerular state; the resulting pseudotime was derived from the slingPseudotime function. Representative units were validated by clinical researchers using H&E staining to correspond to Normal-appearing, immune cell–infiltrated, and cellular crescentic stages. For each glomerulus, a predicted radius was computed as the 95th percentile of distances from the unit centroid; cells within 2 × the radius were defined as peri-glomerular cells. Genes associated with the trajectory were identified by computing per-gene correlations with pseudotime (Pearson) and selecting the top 31 genes among those with positive correlations after Benjamini–Hochberg adjustment (adjusted P value < 0.05). Heatmaps were generated with pheatmap using row-wise z-scoring. We plotted smoothed trajectories of population proportions across pseudotime using the geom_smooth function with method = “loess” (span = 0.75) and standard error (se = TRUE) displayed. For descriptive visualization, arrows and dashed vertical lines were added to indicate phases along pseudotime.

### PTC damage trajectory analysis

We used Monocle3 (version 1.3.7) to analyze the PTC damage trajectory. A monocle3 cell_data_set was constructed directly from the Seurat Xenium counts (GetAssayData(slot = “counts”)), followed by dimensionality reduction with reduce_dimension after preprocess_cds(num_dim = 50), clustering with cluster_cells (Louvain), and trajectory inference with the learn_graph function (use_partition = FALSE, close_loop = FALSE, learn_graph_control = list(ncenter = 200)). We then set normal PTC as the root using the order_cells function to infer lineage progression associated with PTC damage. For each PTC, cells within a 50 µm radius were defined as pericellular neighbors, and their accumulation along pseudotime was analyzed. Changes in cell type abundance across pseudotime were fitted using geom_smooth with the loess method, span = 0.75, with standard error displayed (se = TRUE). UMAP reduction in Monocle3 was run with umap.n_neighbors = 8 and 15, and umap.min_dist = 0.1, and pseudotime visualizations were generated with the plot_cells function.

### Transcription factor and module score analysis

Gene signature scores were computed using AddModuleScore with mCAF (Cords et al., 2023), SAM (Ramachandran et al., 2019), GOBP_T_HELPER_1_CELL_DIFFERENTIATION (Subramanian et al., 2005), and GOBP_T_HELPER_17_CELL_DIFFERENTIATION (Subramanian et al., 2005) gene sets, using ctrl = 100. Transcription factor activity scores of TP53 were calculated using the ENCODE and ChEA Consensus TFs database (Lachmann et al., 2010).

### Cell–cell interaction analysis

We used CellChat (version 2.1.2) to analyze cell–cell interactions, which utilizes the x and y coordinates of cells available from spatial transcriptomic datasets to infer cell–cell communication, considering spatial proximity to estimate the probability of communication. We used the computeCommunProb function with an interaction range of 100 µm and scale.distance = TRUE, referencing CellChatDB.human to calculate the probability of spatial cell–cell interactions. Normal, Early_Damaged, and Terminal_Damaged PTC populations were set as “sender,” and immune cell populations were set as “receiver.” Chemokine-related interactions were selected, and the top 35% by probability were visualized using the netVisual_chord_gene function.

### Differential abundance analysis

Differential abundance analysis of lymphocytes, DC/macrophages, and neutrophils between the Damaged_Proximal and Normal_Proximal niches was performed using miloR (Dann et al., 2022). A k-nearest neighbor graph was constructed with buildGraph using precomputed supervised PCA coordinates with k = 10 and 15 PCs. Cell neighborhoods were then defined with makeNhoods based on graph connectivity. For computational efficiency, 20% of cells were subsampled for each cell type. Differential abundance was tested by fitting a negative binomial generalized linear model to neighborhood cell counts, with TMM normalization to account for differences in total cell numbers across samples. The log_2_ fold change in cell abundance between the two niches was used for visualization, and neighborhoods with FDR < 0.1 were displayed as significantly differentially abundant.

### Cross-niche analysis

To compare immune cells in the Damaged_PTC niche versus the normal niche, we conducted PERMANOVA (vegan::adonis2) on UMAP embedding coordinates separately for each cell type, using adonis2(coords ∼ niche_status, permutations = 999), and reported R2 as the effect size on UMAP embedding coordinates with strata set to sample ID.

### ELISA

Full-length and truncated human MPO proteins were designed based on the UniProt entry P05164 and synthesized by WuXi Biologics. The design of the truncation constructs was guided by a previously reported study (Erdbrugger et al., 2006), and the truncated fragments were defined according to specific aa regions: L (light chain: aa 164–274), Ha (heavy chain: aa 279–409), Hb (heavy chain: aa 399–519), Hc (heavy chain: aa 510–631), and Hd (heavy chain: aa 622–745). MPO proteins were diluted in bicarbonate buffer and added to each well in a volume of 100 μl, followed by overnight incubation at 4°C. Subsequently, the wells were washed three times. Blocking buffer (#T7131A; Takara) was then added in a volume of 200 μl per well, and the plate was agitated gently at room temperature for 1 h. Patient serum samples, diluted 100-fold in blocking buffer, were added to each well in a volume of 100 μl, and the plate was agitated gently at room temperature for an additional 3 h. Goat anti-human IgG Fc secondary antibody (#18820; Invitrogen) was applied, and the plate was agitated gently at room temperature for 1 h. This was followed by five wash cycles. For colorimetric detection, 1-step PNPP substrate (#37621; Thermo Fisher Scientific) was added in a volume of 100 μl to each well. The plate was then agitated gently in the dark at room temperature for 30 min. The reaction was terminated by adding 2N NaOH, and the absorbance was measured at 405 nm.

### Purification of mouse anti-MPO monoclonal antibodies

C57BL/6J MPO-deficient mice were purchased from Jackson Laboratories. In the first campaign, 20 MPO knockout mice were immunized with recombinant mouse MPO protein (WEHI-3 cell derived). Immunization was performed by intraperitoneal injection of 20 µg protein emulsified in CFA on day 0, followed by 10 µg protein in IFA on weeks 3 and 5. Mice were euthanized at week 6. Elevated serum anti-MPO antibody titers were confirmed by ELISA as previously reported (Xiao et al., 2002). B cells were then sorted from splenocytes by flow cytometry: splenocytes were mixed with biotinylated mouse MPO, followed by reaction with streptavidin-FITC and anti-mouse IgG-PE antibodies. PE- and FITC-positive B cells were sorted and plated in 384-well plates, one cell per well, using the limited dilution technique. Antibodies that bound to MPO were screened by ELISA using cell supernatants. Briefly, recombinant mouse MPO protein was immobilized on ELISA plates and cell supernatants were added. Anti-mouse IgG-biotin antibody (#1030-08; SouthernBiotech) was diluted to 1 µg/ml and used as the primary antibody. Streptavidin-alkaline phosphatase conjugate (#11089161001; Roche) was used as the secondary antibody, and luminescence was measured using a SpectraMAX M2 microplate reader with an emission maximum at 461 nm. After screening 88 specific B cell clones, genetic cloning of anti-MPO antibodies was performed. The DNA segments encoding the variable regions of their heavy and light chains were inserted into the pEF-BOS-based expression vector carrying the constant region of mouse IgG2a heavy or light chain. The recombinant IgG was produced in FreeStyle 293-F cells and purified by Protein A-Sepharose. These cloning procedures were performed using an established, previously described method (Sakuragi et al., 2021). In the second campaign, MPO immunization was performed by intradermal injection of 100 µg protein emulsified in CFA on day 0, followed by 100 µg protein in IFA on weeks 2 and 4. At week 8, 20 µg MPO was injected intravenously. Mice were euthanized the next day. B cell sorting, culture, and genetic cloning of anti-MPO antibodies were performed in a similar manner to the first campaign.

### Cryo-EM sample preparation and data collection

For structural determination by cryo-EM, complexes of recombinant mouse MPO (catalog #3667-MP-250; R&D Systems) with anti-MPO Fab fragments were prepared. The Fab fragments were generated from anti-MPO monoclonal antibodies (mAb-A24, mAb-A46, and mAb-B88) by limited proteolysis using the FabULOUS enzyme (Genovis AB). The digestion products were subjected to a two-step purification process: Fc fragments were first removed using a Protein A affinity column (MabSelect SuRe, Cytiva), followed by size-exclusion chromatography on a Superdex 75 Increase 10/300 column (Cytiva) in 20 mM HEPES (pH 7.5) and 300 mM NaCl to isolate pure Fab fragments for complex formation with MPO. The MPO–mAb-A24–mAb-A46 complex was prepared by mixing the components at a molar ratio of 1:2:2 (MPO: mAb-A24 Fab: mAb-A46 Fab). To suppress protein aggregation, the complex was PEGylated with MS(PEG)8 (Thermo Fisher Scientific) (250 mM in DMSO) at a final concentration of 1 mM for 1 h on ice. The reaction was quenched with 1–10th volume of 1 M Tris-HCl (pH 8.0). PEGylation of the protein complex was confirmed by SDS-PAGE analysis, which showed band shifts consistent with the increased molecular weight of the PEGylated proteins. To remove excess PEG and exchange the buffer, the MPO–mAb-A24–mAb-A46 complex was purified by size-exclusion chromatography using 20 mM HEPES (pH 7.5) and 200 mM NaCl as the running buffer. Fractions containing the complex were collected and concentrated to 5.3 mg/ml using an Amicon concentrator (MWCO 30 kDa). Similarly, an MPO–mAb-A46–mAb-B88 complex was prepared by mixing the components at a molar ratio of 1:2:2 (MPO: mAb-A46 Fab: mAb-B88 Fab) under the same buffer conditions. This complex underwent the same PEGylation, purification, and concentration procedures and was concentrated to 5.1 mg/ml as described for the MPO–mAb-A24–mAb-A46 complex.

For cryo-EM analysis, the MPO–mAb-A24–mAb-A46 complex and MPO–mAb-A46–mAb-B88 were prepared at 5.1 mg/ml and 5.0 mg/ml, respectively, in 20 mM HEPES (pH 7.5), 200 mM NaCl, and 0.03% DDM. Quantifoil grids (Cu R0.6/1.0) were glow-discharged at 7 Pa and 10 mA for 10 s using a JEC-3000FC (JEOL). The protein complex (3 μl) was applied to glow-discharged grids, blotted for 2 s at 4°C and 100% humidity, and plunge-frozen in liquid ethane using a Vitrobot Mark IV (Thermo Fisher Scientific). Data collection was performed on an in-house Glacios microscope (Thermo Fisher Scientific) equipped with a Selectris-X energy filter and Falcon 4i detector, operating at 200 kV at Chugai Pharmaceutical, Kamakura Research Center. EER movies were collected at a physical pixel size of 0.887 Å/pixel with a total dose of 60 electrons/Å^2^. Detailed parameters used for cryo-EM data acquisition are provided in Table S4.

### Cryo-EM data processing and model building

EER files were subjected to beam-induced motion correction using RELION 5.0.0 (Scheres, 2012), and contrast transfer function (CTF) parameters were determined using CTFFIND4 (Rohou and Grigorieff, 2015). All subsequent particle picking, 2D classification, 3D classification, and initial refinement steps were performed using cryoSPARC version 4.6.2 (Punjani et al., 2017). For structural analysis of the MPO–mAb-A24–mAb-A46 and MPO–mAb-A46–mAb-B88 complexes, 18,496 and 7,115 movies were collected, respectively. Initial particle selection was performed using the blob picker in cryoSPARC. Selected particles underwent 2D classification, ab initio reconstruction, and non-uniform refinement to generate preliminary low-resolution 3D maps. These maps served as references for template-based particle picking to expand the dataset. Subsequently, ab initio reconstruction was performed with 100,000 particles to generate five decoy 3D maps. Heterogeneous refinement was conducted using six initial volumes: the five decoy maps from ab initio reconstruction and one 3D map obtained from blob-picked particles. The particle subset from the highest-resolution class was exported to RELION for Bayesian polishing. The polished particles were reimported into cryoSPARC and subjected to non-uniform refinement, yielding final reconstructions at 2.95 Å resolution for both the MPO–mAb-A24–mAb-A46 and MPO–mAb-A46–mAb-B88 complexes. Initial models of both complexes were built using ModelAngelo (Jamali et al., 2024). All models were built and refined in ISOLDE (Croll, 2018) and Coot (Emsley et al., 2010), followed by real-space refinement in Phenix (Liebschner et al., 2019). Model geometry and map–model validation were assessed using Phenix, with detailed statistics provided in Table S5. All graphical representations were prepared using UCSF ChimeraX version 1.7.1 (Meng et al., 2023).

### Quantification of neutrophil-generated ROSs

Wild-type mouse bone marrow neutrophils were isolated by the MACS method using the Neutrophil Isolation Kit (cat. no. 130-097-658; Miltenyi Biotec). Isolated neutrophils were resuspended in RPMI1640 medium at a concentration of 4.0 × 10^6^ cells/ml. Extracellular ROSs were quantified using the Fluorimetric Hydrogen Peroxide Assay Kit (Cat No. MAK165; Sigma-Aldrich). Master mix was pre-applied at 50 μl/well. Neutrophils with indicated stimuli were added at 1.0 × 10^5^ cells per well. Fluorescence was measured every minute for 2 h at an excitation wavelength of 540 nm and an emission wavelength of 590 nm with the microplate reader preheated to 37°C.

### Quantification of NETs

8–10-wk-old mice were administered an initial intraperitoneal injection of TNF-α (0.8 µg/mouse). 2 h following the TNF-α injection, an additional intraperitoneal injection of anti-MPO antibodies or control anti-KLH antibody was given. PECs were collected 2 h later by lavage using PBS containing 2 mM EDTA. The cells were then centrifuged and pelleted. The subpopulations of Ly6G-positive and SYTOX Green-positive cells were identified and quantified, indicative of NETosis.

### Development of a monoclonal anti-MPO antibody–induced glomerulonephritis model

8–14-wk-old 129S6 wild-type mice were used for antibody-induced vasculitis experiments. On day 0, mice received an intraperitoneal injection of Zymosan A (0.5 mg or 0.1 mg, as indicated), followed by intravenous injections of a monoclonal anti-MPO antibody cocktail diluted in PBS. Each antibody in the cocktail was administered at a total dose of 15 mg/kg. In the first set of experiments, injections were given twice weekly, whereas in the second set, once weekly. All mice were euthanized at the end of the experimental period (week 3). Disease severity was assessed by proteinuria measurements, and kidneys were harvested and fixed in 10% neutral-buffered formalin. The frequency of glomerular crescent formation was evaluated by expert renal pathologists at Eurofins Genetic Lab (Sapporo, Japan). Blood and urine samples were collected and analyzed for biochemical markers using a TBA-120FR analyzer (Canon). Urine samples with gross hematuria caused by bladder puncture were excluded from analysis. From each mouse, 5-μm-thick sections were cut from kidney FFPE blocks and processed for in situ profiling on the 10x Genomics Xenium platform using the same protocol as for human specimens. The resulting datasets were preprocessed and analyzed in R using the same pipelines as those applied to the human samples.

### Therapeutic intervention with one-armed anti-MPO antibodies in the passive transfer glomerulonephritis model

The passive transfer glomerulonephritis model was established as previously described (Xiao et al., 2002); 8–14-wk-old MPO-deficient mice were pre-immunized with mouse MPO in CFA at week 0, followed by IFA at weeks 2 and 4, and received an intravenous MPO boost at week 6. Splenocytes were harvested and transferred into 8–14-wk-old Rag2-deficient recipient mice at a concentration of 1 × 10^8^ cells per mouse via intravenous injection. On the day of splenocyte transfer, recipient mice also received an intraperitoneal injection of 0.1 mg Zymosan A. These mice were euthanized at week 4. One-armed IgG2a antibodies, engineered to correspond to pathogenic anti-MPO antibodies, were generated by Chugai Pharma. Antibody constructs were cloned into an *Escherichia coli* expression vector, expressed, and purified by Protein A affinity chromatography after microfluidization. In therapeutic experiments, one-armed antibodies were administered to mice following splenocyte transfer. Treatment efficacy was assessed by proteinuria, renal histopathology (crescent formation and fibrosis), and biochemical markers as described above.

### Online supplemental material

Fig. S1 shows gene expression profiles of human kidney tissues, related to Fig. 1. Fig. S2 shows pseudotime trajectories in distant peri-glomerular regions and clinical correlations of glomerular-region cell populations, related to Fig. 3. Fig. S3 shows subset analysis of PEC and loop of Henle cell populations, related to Fig. 4. Fig. S4 shows characterization and functional assessment of anti-MPO antibodies, related to Fig. 6. Fig. S5 shows cell annotation and spatial niche organization in anti-MPO antibody–treated mouse kidneys, related to Fig. 7. Table S1 shows characteristics of eight patients with MPO-ANCA–associated glomerulonephritis recruited for Xenium. Table S2 shows highly expressed genes in fibroblast and macrophage subsets identified by DEG analysis. Table S3 shows characteristics of 23 patients with MPA recruited for assessing MPO-ANCA epitopes. Table S4 shows a list of aa residues on MPO identified as epitopes for each monoclonal antibody based on cryo-EM structural analysis. Table S5 shows cryo-EM data collection and validation statistics for MPO–antibody complexes.

## Supplementary Material

Table S1 shows characteristics of eight patients with MPO-ANCA–associated glomerulonephritis recruited for Xenium.

Table S2 shows highly expressed genes in fibroblast and macrophage subsets identified by DEG analysis.

Table S3 shows characteristics of 23 patients with MPA recruited for assessing MPO-ANCA epitopes.

Table S4 shows a list of aa residues on MPO identified as epitopes for each monoclonal antibody based on cryo-EM structural analysis.

Table S5 shows cryo-EM data collection and validation statistics for MPO–antibody complexes.

## Data Availability

Spatial transcriptomic data are available at Zenodo with DOI https://doi.org/10.5281/zenodo.18229261. Donor-specific spatial maps and associated visualization files are available at Figshare (https://doi.org/10.6084/m9.figshare.32725488). scRNA-seq data are available at the Genomic Expression Archive with accession code E-GEAD-867. Cryo-EM structures have been deposited in the RCSB Protein Data Bank (PDB) and the Electron Microscopy Data Bank (EMDB). The structure of mouse MPO in complex with Fab fragments of mAb-A24 and mAb-A46 has been deposited under accession codes PDB 21RN and EMDB EMD-67945. The structure in complex with Fab fragments of mAb-A46 and mAb-B88 has been deposited under accession codes PDB 21RT and EMDB EMD-67951. All data are included in the online supplemental material or available from the authors, as are unique reagents used in this article. The raw numbers for charts and graphs are available in the source data files whenever possible. Experimental protocols and the data analysis pipeline were developed with reference to the official documentation for 10x Xenium, BD Rhapsody, and Seurat. The analysis steps, functions, and parameters used are described in detail in the Materials and methods section. Custom code used in the paper is available at the following website (https://github.com/KeiNishim/MPA_Spatial) and was archived on Zenodo with DOI https://doi.org/10.5281/zenodo.18230533.
